# Influence of *DNMT* Genotype on Global and Site Specific DNA Methylation Patterns in Neonates and Pregnant Women

**DOI:** 10.1371/journal.pone.0076506

**Published:** 2013-10-01

**Authors:** Catherine Potter, Jill McKay, Alexandra Groom, Dianne Ford, Lisa Coneyworth, John C. Mathers, Caroline L. Relton

**Affiliations:** 1 Human Nutrition Research Centre, Institute of Genetic Medicine, Newcastle University, Newcastle upon Tyne, Tyne and Wear, United Kingdom; 2 Human Nutrition Research Centre, Institute of Health and Society, Newcastle University, Newcastle upon Tyne, Tyne and Wear, United Kingdom; 3 Human Nutrition Research Centre, Institute for Cell and Molecular Biology, Newcastle University, Newcastle upon Tyne, Tyne and Wear, United Kingdom; 4 Human Nutrition Research Centre, Institute for Ageing and Health, Newcastle University, Newcastle upon Tyne, Tyne and Wear, United Kingdom; 5 MRC Integrative Epidemiology Unit, School of Social and Community Medicine, University of Bristol, Bristol, United Kingdom; Universität Stuttgart, Germany

## Abstract

This study examines the relationship between common genetic variation within DNA methyltransferase genes and inter-individual variation in DNA methylation. Eleven polymorphisms spanning *DNMT1* and *DNMT3B* were genotyped. Global and gene specific (*IGF2, IGFBP3, ZNT5*) DNA methylation was quantified by LUMA and bisulfite Pyrosequencing assays, respectively, in neonatal cord blood and in maternal peripheral blood. Associations between maternal genotype and maternal methylation (n ^≈^ 333), neonatal genotype and neonatal methylation (n ^≈^ 454), and maternal genotype and neonatal methylation (n ^≈^ 137) were assessed. The findings of this study provide some support to the hypothesis that genetic variation in DNA methylating enzymes influence DNA methylation at global and gene-specific levels; however observations were not robust to correction for multiple testing. More comprehensive analysis of the influence of genetic variation on global and site specific DNA methylation is warranted.

## Introduction

DNA methylation is an epigenetic mechanism that plays an important role in gene expression [1], tissue differentiation [2] and genomic imprinting [3]. Addition of methyl groups to the 5’ position on cytosine residues in DNA is mediated by the family of DNA methyltransferase (DNMT) proteins, including DNMT1, DNMT3A, DNMT3B and DNMT3L. DNMT1 plays a major role in the maintenance of methylation patterns, exhibiting a higher level of activity on hemi-methylated, compared with unmethylated, DNA [4]. *De novo* methylation is catalysed by DNMT3A and DNMT3B. DNMT3L is also involved in *de novo* methylation through binding to DNMT3A and DNMT3B and via its ability to recognise the modification status of other epigenetic marks including histone proteins [5].

Whilst numerous studies have implicated environmental and lifestyle factors in inter-individual variation in DNA methylation patterns in humans [6,7], considerably less is known about the role of common genetic variation [8-10]. Longitudinal analysis of DNA methylation, where blood samples were taken approximately 16 years apart, found familial clustering in the degree of change in DNA methylation levels over time [11], suggesting that the maintenance of epigenetic patterns is partly under genetic control. Genetic determinants of DNA methylation in individuals may therefore make an important contribution to variance in this trait and potentially to a range of phenotypes including disease risk.

In animal models, genetic manipulation of *DNMT* genes demonstrates clearly their essential role in development with knockouts of *DNMT1* and *DNMT3B* being embryonic lethal in mice [12,13]. A number of human monogenic disorders result from mutations in these genes [14]. For example, mutations in exon 20 and 21 of *DNMT1* result in the autosomal dominant hereditary sensory and autonomic neuropathy type 1 while *DNMT3* mutations result in the recessive immunodeficiency centromeric instability facial syndrome 1. In addition, common genetic variants spanning the *DNMT* genes have been associated with several complex phenotypes. For example, there are associations between *DNMT3B* genotype, breast cancer susceptibility, lung cancer susceptibility and prostate cancer progression [15-17]. Although the exact mechanisms through which these *DNMT* mutations cause the corresponding pathogenic phenotypes are not fully understood, they have been associated with altered global DNA methylation levels [14,18]. Furthermore, single nucleotide polymorphisms (SNPs) in *DNMT* genes have been associated with functional variation i.e. altered activity of the encoded protein [16,19,20], which is postulated to cause aberrant methylation patterns. However, most studies investigating the effects of *DNMT* gene polymorphisms on downstream DNA methylation patterns have been in the context of disease phenotypes rather than a general population based cohort.

We hypothesised that genetic variants within genes responsible for maintenance of DNA methylation (*DNMT1*) and for *de novo* DNA methylation (*DNMT3B*) alter methylation profiles at specific loci and, more so, influence levels of global DNA methylation. We tested this hypothesis in samples taken from adult women and their neonatal offspring from the North Cumbria Community Genetics Project (NCCGP) [21]. Since the influence of environmental and lifestyle exposures on DNA methylation patterns may increase over time [22] such potential confounding may be minimised in the neonates. In addition, given the evidence for inter-generational influences on DNA methylation patterns [23,24] we also considered the secondary hypothesis that maternal *DNMT* genotype influence DNA methylation patterns in their neonatal offspring.

## Materials and Methods

### Study population

Peripheral blood DNA samples were available for 333 mothers along with cord blood DNA samples from 454 neonates, including 137 mother-child pairs. These subjects were recruited as part of the NCCGP, between 1996 and 2003, at a single maternity unit in West Cumbria, UK [21]. Mothers completed a detailed questionnaire and provided blood samples during their first antenatal clinic (mean (SD) gestation = 11.3 (5.4) weeks). Cord blood from neonates was collected at birth (mean (SD) gestation = 39.5 (1.4) weeks). Written informed consent was obtained from all participating mothers recruited during pregnancy. The consent obtained included use of their own biological samples and those of their child (including DNA) for epidemiological studies. Ethical approval to undertake this study was obtained from the Cumbria and Lancashire Local Research Ethics Committee.

### Genotype analysis for DNMT1 and DNMT3B

Eleven SNPs spanning the *DNMT1* and *DNMT3B* genes were genotyped across all study samples by K-Bioscience using their proprietary KasPar system (www.kbioscience.co.uk/). These included 2 pairwise tagging SNPs for *DNMT1* (rs2290684, rs2241531) and 8 pairwise tagging SNPs for *DNMT3B* (rs6119954, rs992472, rs2424928, rs2424932, rs6058897, rs437302, rs406193) as described previously by Cebrian et al [15]. In addition, two *DNMT3B* SNPs (rs1569686, rs2424913) located within the promoter/5' gene region and previously associated with altered promoter activity were selected for investigation [16,19,25,26]. Polymorphisms demonstrating genotyping success rates less than 80% and minor allele frequencies (MAF) less than 5% were removed prior to analysis. Hardy Weinberg Equilibrium (HWE) was assessed for each SNP; those demonstrating deviations from HWE (P < 0.05) were removed prior to analysis.

### LUMA assay to determine global DNA methylation

Genomic DNA methylation was measured as part of our previously published study [27] and was available for a subset of individuals in the current analysis (Table 1). The luminometric methylation assay (LUMA) protocol has been described in detail previously [27,28]. Briefly, 200ng DNA was incubated for 4h at 37°C with 1) methylated (EcoRI plus MspI) and 2) unmethylated (EcoRI plus HpaII) restriction enzyme sets in 20µl volume reactions containing 5 units of each enzyme (New England Biolabs) and 2µl of Tango buffer (Fermentas). Digests were carried out in triplicate for each sample and analysed by Pyrosequencing on a Pyromark™ MD system. Data are presented as a methylation ratio (defined as (HpaII/EcoRI)/(MspI/EcoRI)) with a higher ratio indicative of less methylated DNA.

**Table 1:** Global and loci specific methylation levels and correlation.

	Mothers		Average Correlation^†^		Infants		Average Correlation^†^	
Methylation locus	Success Rate*	**Median (25%, 75%) Methylation**	**rho**	***p***	Success Rate*	**Median (25%, 75%) Methylation**	**rho**	***p***
**Global, ratio**	213/286 (74%)	0.32 (0.27, 0.44)			320/356 (90%)	0.36 (0.31, 0.41)		
***IGF2* Site1**	311/326 (95%)	42.47 (38.62, 45.32)			405/423 (96%)	45.40 (41.85, 48.15)		
***IGF2* Site2**	309/326 (95%)	49.64 (46.34, 52.91)	0.81	<0.001	403/423 (95%)	51.98 (49.56, 54.78)	0.82	<0.001
***IGF2* Site3**	304/326 (93%)	47.30 (44.38, 49.86)			409/423 (97%)	50.11 (47.20, 52.28)		
***IGF2* Mean**	326	46.38 (43.31, 48.97)			423	49.35 (46.43, 51.55)		
***IGFBP3* Site1**	244/244 (100%)	5.56 (3.94, 6.91)			322/326 (99%)	4.85 (4.03, 5.75)		
***IGFBP3* Site2**	244/244 (100%)	6.23 (5.18, 7.54)			323/326 (99%)	5.95 (5.30, 6.72)		
***IGFBP3* Site3**	244/244 (100%)	4.98 (4.15, 6.16)	0.77	<0.001	322/326 (99%)	4.43 (3.92, 5.18)	0.64	<0.001
***IGFBP3* Site4**	242/244 (99%)	8.25 (7.04, 9.51)			324/326 (99%)	7.42 (6.55, 8.24)		
***IGFBP3* Site5**	243/244 (100%)	6.17 (4.99, 7.52)			319/326 (98%)	6.55 (5.64, 7.64)		
***IGFBP3* Mean**	244	6.25 (5.12, 7.36)			326	5.83 (5.24, 6.59)		
***ZNT5* Site2**	215/218 (99%)	90.00 (79.50, 95.00)	0.26	<0.001	341/350 (97%)	94.50 (88.00, 97.00)	0.24	<0.001
***ZNT5* Site3**	212/218 (97%)	92.00 (85.00, 95.50)			342/350 (98%)	96.25 (89.50, 99.00)		
***ZNT5* Site4**	173/218 (79%)	100 (97.50, 100)	Excluded		304/350 (87%)	100 (98.00, 100)	Excluded	
***ZNT5* Site5**	139/218 (64%)	69.50 (64.00, 85.50)	Excluded		263/350 (75%)	70.50 (63.50, 90.00)	Excluded	
***ZNT5* Site6**	48/218 (22%)	84.00 (79.75, 86.00)	Excluded		161/350 (46%)	80.50 (74.50, 83.50)	Excluded	

* Global and loci specific methylation data was not available for all subjects. ^†^ Correlation assessed by nonparametric Spearman’s rank.

### Bisulfite Pyrosequencing for candidate gene DNA methylation analysis

In addition to global methylation three candidate genes, namely *IGF2*, *IGFBP3* and *ZNT5*, were selected for analysis. These genes were selected as they represent three contrasting degrees of methylation; *IGF2* is an imprinted locus with mean methylation ~50%, *IGFBP3* is constitutively methylated at low levels (~5%) and *ZNT5* is constitutively methylated at high levels (~90%). Furthermore, we have previously shown that DNA methylation levels at these three loci are variable and influenced by a combination of genetic and environmental factors [27].

Methylation levels at the three candidate loci were measured using bisulfite Pyrosequencing as part of our previously published study [27] and made available for the current analysis. Table 1 reports the number of CpG sites and the number of samples assessed for the three loci. Pyrosequencing and quality control (QC) methods were described in detail previously [27]. Briefly, bisulfite conversion of 2µg of genomic DNA was performed using EZ DNA Methylation Gold™ kit (Zymo Research) following the manufacturer’s protocol. Quantitative bisulphite Pyrosequencing (Qiagen, UK) with Pyro Q-CpG™ Software (version 1.0.6.) was subsequently used to determine the percentage methylation at individual CpG sites within the differentially methylated region (DMR) of *IGF2* (NG_008849.1; 6098-6375), the *IGFBP3* promoter (NT_007819.17; 45951336-45951104) and the *ZNT5* promoter (NT_006713.15; 18983340-18983714). 0.2µg of bisulfite treated DNA was added to the first PCR reaction with 12.5µl Hot Star Taq mastermix (Qiagen) and optimised primer concentrations. Complete primer and PCR conditions are described in Table S1 in File S1. Subsequent sequencing reactions followed the manufacturer’s protocol. Assays were assessed for amplification bias and reliability as described previously [27,29]. Zero and 100% *in vitro* methylated controls were run routinely alongside samples as internal controls.

CpG sites with poor success rates across the study population (<80% success) were removed prior to analysis. Similarly, CpG sites demonstrating extreme measures of methylation with limited variability across the study population (median methylation = 0% or 100%) were dropped prior to analysis. The genotypic and methylation data can be made available upon request to the authors.

### Data analysis

Linkage disequilibrium (LD) between genetic variants was assessed using Haploview software [30]. Correlation in methylation levels at individual CpG sites spanning the 3 candidate loci was assessed using non-parametric Spearman’s rank correlation. Those loci demonstrating at least modest correlation (rho > 0.6) across individual CpG sites were also analysed using the mean percentage methylation for that locus. Non-parametric tests for trend were performed to explore relationships between methylation levels and SNPs under an additive model. Polymorphisms with low MAF (i.e. <0.15) were analysed under a dominant model, with respect to the minor allele, using Kruskal Wallis tests. Comparisons between methylation levels in maternal-infant pairs were made by non-parametric Spearman’s rank correlation and Wilcoxon signed-rank tests. Uncorrected *p*-values are presented throughout with p ≤ 0.05 considered statistically significant. Corrections were applied post hoc when interpreting the results to assess whether nominally significant observations were robust to correction for multiple testing. Bonferroni as well as Benjamini and Hochberg corrections were applied in this context. All statistical analyses were performed in Stata (version 10, Statacorp).

### Power of the study to detect associations between DNMT genotype and DNA methylation

With 333 maternal samples, there was 94% power to detect a 1.4 fold change in global methylation assuming an additive model with a 5% MAF at the 0.05 significance level. This power calculation was based on data reported previously [27] in which the mean (SD) methylation ratio across maternal samples was 0.46 (0.3). Under the same model there was 84% power to detect an absolute change of 4% in *IGF2* methylation, 87% power for an absolute change of 4% in *IGFBP3* methylation and 81% power for an absolute change of 6% in *ZNT5* methylation. Again these calculations were based on previously reported data in which the mean (SD) methylation percentages for *IGF2*, *IGFBP3* and *ZNT5* were 45 (8)%, 7 (7)%, 90 (12%), respectively [27]. There was greater power to detect the same effects in the larger neonate cohort (n=454). Power calculations were performed in Quanto [31,32].

## Results

### Cohort characteristics

Peripheral blood DNA samples were available for 333 mothers along with cord blood DNA samples from 454 neonates. The average age at the time of sample collection from the mothers was 29 years and 52% of the neonates were male (see Table S2 in File S1). In addition, these samples comprised 137 mother-child pairs. These pairs were representative of their larger sample groups (see Table S2 in File S1). SNPs mapping to the *DNMT1* and *DNMT3B* genes were genotyped across all samples. Pyrosequencing data was previously generated on smaller subsets of these samples (Table 1).

### Genotype data across the DNMT1 and DNMT3B genes

**Figure 1:**
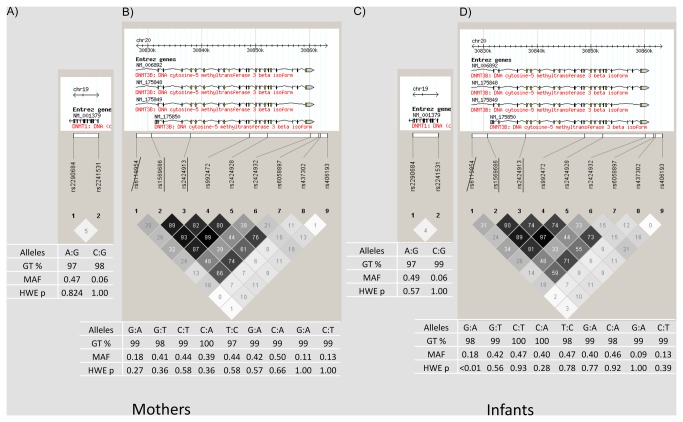
Linkage disequilibrium and SNP frequencies across DNMT genes in mothers and infants. Alleles = Major: Minor, GT % = genotyping success rate (mothers total N = 333, infants total N = 454), MAF = minor allele frequency, HWE p = Hardy Weinberg Equilibrium p-value. Single underscore = putative functional SNPs; strikethrough = excluded from analysis.

Eleven SNPs, with MAF >0.05, were genotyped successfully in both neonates and adult women (Figure 1). Three of these SNPs (*DNMT1* rs2241531, *DNMT3B* rs437302 and rs406193) had relatively low MAF (<0.15) and underwent exploratory analyses using a dominant rather than an additive model. One *DNMT3B* SNP (rs6119954) did not conform to HWE (p = 0.003) in the neonate group and was excluded from further analysis. The two putative functional SNPs rs1569686 and rs2424913 demonstrated strong LD (r^2^>0.8) with the *DNMT3B* rs992472 and rs2424928 tagging SNPs (Figure 1). In total, 10 SNPs were taken forward for analysis.

### Correlation between CpG sites within the IGF2, IGFBP3 and ZNT5 genes

In addition to global methylation, methylation of 3 CpG sites in the DMR of the imprinted gene *IGF2* and 5 CpG sites in both the *IGFBP3* and *ZNT5* promoter regions was quantified (Table 1). CpG site 4 in *ZNT5* was very highly methylated with little inter-individual variation (median methylation = 100%) and was excluded from further statistical analysis. CpG sites 5 and 6 in *ZNT5* were dropped from further statistical analysis due to poor success rates (<80%) in both maternal and neonate samples. The remaining CpG sites (3 within *IGF2*, 5 within *IGFBP3* and 2 with *ZNT5*) demonstrated high success rates in both maternal and neonate samples (Table 1) and were taken forward for analysis. In addition, there were modest to strong correlations (average rho: 0.64-0.82) demonstrated between methylation levels at the CpG sites mapping to the *IGF2* and the *IGFBP3* loci in both mothers and infants (Table 1). Hence, mean methylation levels across these two loci were calculated and these means were also used in further analyses. Weaker correlations (rho<0.6) were found between methylation levels of the CpG sites measured within the *ZNT5* locus therefore only methylation values for individual CpG sites were assessed further. Measurement of global DNA methylation was less successful in the maternal samples (74%) compared to that in the neonate samples (90%). However, we chose to retain this measure for analysis but acknowledge that any findings must be viewed with caution.

### Predictors of maternal methylation

Table 2 summarises data for those variables for which there were statistically significant (p≤0.05) associations between maternal genetic factors and maternal methylation levels. See Table S3 in File S1 for all results. The minor G allele of the *DNMT1* SNP rs2290684 was associated with increased methylation of CpG site 3 in *IGF2* following an additive model. Similar trends were demonstrated at other *IGF2* CpG sites, including the mean methylation across all CpG sites measured, but these did not reach the p≤0.05 threshold for statistical significance (Table S3 in File S1). Rs2290684 was also associated with *ZNT5* CpG site 3 methylation levels.

**Table 2:** Significant associations between methylation and genetic predictors.

			**AA**		**Aa**		**aa**		**Additive^†^**	
**SNP**	**Methylation Locus**	**Alleles**	**N**	**Median (25%, 50%) Methylation**	**N**	**Median (25%, 50%) Methylation**	**N**	**Median (25%, 50%) Methylation**	**Z**	***p***
**Maternal**	**Maternal**									
*DNMT1* rs2290684	*IGF2* Site 3	A:G	81	46.25 (43.87, 48.81)	150	47.46 (44.59, 49.59)	64	48.07 (44.37, 50.73)	2.23	0.026
*DNMT1* rs2290684	*ZNT5* Site3	A:G	59	91.00 (81.50, 94.00)	102	93.50 (86.50, 96.50)	48	93.50 (88.00, 95.75)	2.05	0.041
**Infant**	**Infant**									
*DNMT1* rs2290684	*IGFBP3* Site3	A:G	79	4.26 (3.74, 5.03)	167	4.39 (3.89, 4.94)	69	4.66 (4.09, 5.64)	2.45	0.014
*DNMT1* rs2290684	*IGFBP3* Site5	A:G	79	6.32 (5.40, 7.45)	165	6.63 (5.73, 7.56)	68	6.90 (5.97, 8.05)	2.09	0.036
*DNMT1* rs2241531^ɸ^	*IGFBP3* Site5	C:G	280	6.59 (5.74, 7.69)	37	5.75 (4.18, 7.15)	0	-	5.00	0.025
*DNMT3B* rs1569686	*IGFBP3* Site5	G:T	107	6.32 (5.61, 7.24)	158	6.68 (5.58, 7.68)	53	6.83 (5.96, 8.12)	2.03	0.042
*DNMT3B* rs2424913	*IGFBP3* Site3	C:T	90	4.29 (3.74, 4.85)	165	4.49 (4.00, 5.29)	66	4.53 (4.03, 5.67)	2.50	0.012
*DNMT3B* rs2424913	*IGFBP3* Site5	C:T	92	6.24 (5.49, 7.22)	160	6.57 (5.62, 7.64)	66	6.83 (5.77, 8.15)	2.23	0.026
*DNMT3B* rs992472	*IGFBP3* Site3	C:A	114	4.30 (3.76, 4.88)	163	4.50 (3.94, 5.41)	45	4.51 (4.09, 5.15)	2.23	0.026
*DNMT3B* rs992472	*IGFBP3* Site5	C:A	115	6.32 (5.61, 7.24)	158	6.65 (5.58, 7.68)	46	7.02 (6.07, 8.18)	2.33	0.020
*DNMT3B* rs2424928	*IGFBP3* Site3	T:C	86	4.30 (3.75, 4.85)	167	4.49 (3.97, 5.29)	65	4.51 (4.03, 5.67)	2.16	0.030
*DNMT3B* rs6058897	*IGFBP3* Site3	C:A	85	4.54 (4.01, 5.29)	166	4.49 (3.97, 5.32)	64	4.29 (3.81, 4.60)	-2.07	0.039
*DNMT3B* rs6058897	*IGFBP3* Site5	C:A	85	6.79 (5.77, 8.12)	161	6.55 (5.62, 7.55)	66	6.20 (5.42, 7.26)	-2.05	0.040
*DNMT3B* rs406193^ɸ^	*IGFBP3* Site3	C:T	248	4.50 (3.99, 5.33)	66	4.29 (3.78, 4.85)	7	4.30 (3.74, 4.54)	0.53	0.021
**Maternal**	**Infant**									
*DNMT1* rs2241531^ɸ^	Global	C:G	81	0.35 (0.30, 0.40)	18	0.39 (0.35, 0.42)	0	-	4.35	0.037
*DNMT3B* rs6058897	Global	C:A	27	0.36 (0.30, 0.39)	49	0.35 (0.30, 0.40)	25	0.39 (0.35, 0.41)	2.21	0.027
*DNMT3B* rs1569686	*IGFBP3* Site1	G:T	37	3.77 (2.77, 5.36)	41	4.61 (3.70, 5.28)	17	5.09 (3.09, 6.41)	1.96	0.050
*DNMT3B* rs1569686	*IGFBP3* Site5	G:T	38	5.99 (3.10, 6.51)	40	6.27 (4.66, 6.89)	17	6.88 (6.21, 8.19)	2.96	0.003
*DNMT3B* rs2424913	*IGFBP3* Site1	C:T	36	3.76 (2.40, 5.20)	40	4.61 (3.65, 5.40)	21	5.09 (4.65, 6.41)	2.34	0.019
*DNMT3B* rs2424913	*IGFBP3* Site5	C:T	37	6.00 (3.10, 6.88)	39	6.23 (4.54, 6.79)	21	6.94 (6.21, 8.22)	3.05	0.002
*DNMT3B* rs2424913	*IGFBP3* Mean	C:T	38	5.35 (4.72, 6.30)	40	5.60 (5.23, 5.92)	21	6.02 (5.24, 6.77)	2.05	0.041
*DNMT3B* rs992472	*IGFBP3* Site1	C:A	40	4.38 (2.79, 5.27)	40	4.63 (3.65, 5.40)	16	5.23 (3.84, 6.60)	1.99	0.046
*DNMT3B* rs992472	*IGFBP3* Site5	C:A	41	6.00 (4.48, 6.88)	39	6.31 (4.54, 7.00)	16	6.80 (6.11, 8.21)	2.64	0.008
*DNMT3B* rs2424928	*IGFBP3* Site1	T:C	35	3.74 (2.03, 5.04)	39	4.61 (3.59, 5.28)	21	5.09 (4.65, 6.41)	2.56	0.011
*DNMT3B* rs2424928	*IGFBP3* Site5	T:C	36	5.99 (2.98, 6.70)	39	6.23 (4.54, 6.79)	21	6.94 (6.21, 8.22)	3.28	0.001
*DNMT3B* rs2424928	*IGFBP3* Mean	T:C	37	5.29 (4.72, 6.27)	39	5.52 (5.22, 5.87)	21	6.02 (5.24, 6.77)	2.26	0.024

† Association between methylation and SNP genotypes was initially tested under an additive model using a non-parametric trend test, unless otherwise stated. ɸ SNPs with low MAF were tested under a dominant model (with respect to the minor allele) only. Alleles=Major: Minor.

### Predictors of infant methylation: infant genotypes

Table 2 summarises data for those variables for which there were statistically significant (p≤0.05) associations between infant genetic factors and infant methylation levels. See Table S4 in File S1 for all results. Increased methylation at CpG sites 3 and 5 of the *IGFBP3* locus was associated with the minor G allele of the *DNMT1* rs2290684 variant, whereas carriers of the minor G allele of rs2241531 had lower methylation at CpG site 5 compared with wild-types (Table 2). Several SNPs within the infants’ *DNMT3B* gene were associated with infant *IGFBP3* methylation (Table 2). In particular, carriage of the minor alleles for the putative functional SNPs (rs1569686, rs2424913) along with the minor alleles of the pairwise tagging SNPs rs992472 and rs2424923 was accompanied by increased methylation at CpG sites 3 and/or 5. The most statistically significant association being demonstrated between the putative functional SNP rs2424913 and *IGFBP3* CpG site 3 (p=0.012). In contrast, carriage of the minor alleles at both rs6058897 and rs406193 were associated with decreased methylation at CpG sites 3 and 5.

### Predictors of infant methylation: maternal methylation and genotypes

Greater variability was demonstrated for measures of global DNA methylation across the maternal samples compared to their paired infant samples (Table 3). Small yet notable differences were observed in methylation levels at CpG sites mapping to *IGF2* and *ZNT5* across the maternal and infant paired samples (Table 3). For both genes methylation levels were consistently higher in infant compared to maternal samples. Nonetheless, there was limited pairwise correlation (rho < 0.6) in methylation levels, both global and loci specific, demonstrated between mothers and their infants within the 137 mother-infant pairs (Table 3). Similarly, there were no observable differences in methylation levels at CpG sites mapping to *IGFBP3* between the mothers and their infant pair.

**Table 3:** Comparison of methylation levels in maternal-infant pairs.

	Median (25%, 75%) Methylation				Mother-Infant Comparison^†^		Mother-Infant Correlation^ɸ^	
Methylation locus	N*	**Mothers**	**Infants**	**Paired Difference**	**z**	***p***	**rho**	***p***
**Global, ratio**	59	0.34 (0.28, 0.62)	0.35 (0.30, 0.39)	0.03 (-0.06, 0.26)	2.48	0.0130	0.18	0.1650
***IGF2* Site1**	115	41.81 (38.95, 45.15)	45.56 (41.42, 47.64)	-2.52 (-6.14, 2.18)	-3.64	0.0003	-0.04	0.6572
***IGF2* Site2**	118	49.22 (46.32, 52.68)	51.97 (48.50, 54.24)	-2.23 (-6.29, 2.45)	-2.86	0.0043	-0.01	0.9566
***IGF2* Site3**	113	46.60 (44.31, 49.94)	49.89 (46.61, 52.22)	-1.93 (-5.49, 1.72)	-3.06	0.0022	0.01	0.9055
***IGFBP3* Site1**	89	4.81 (3.06, 5.88)	4.65 (3.00, 5.39)	0.07 (-1.28, 1.46)	0.52	0.6062	0.30	0.0037
***IGFBP3* Site2**	91	5.73 (4.91, 7.08)	5.68 (5.12, 6.44)	0.10 (-0.92, 1.50)	0.71	0.4750	0.08	0.4239
***IGFBP3* Site3**	90	4.38 (3.60, 5.21)	4.34 (3.82, 4.94)	0.02 (-1.23, 1.13)	0.29	0.7705	0.17	0.1117
***IGFBP3* Site4**	90	7.55 (5.85, 8.63)	7.12 (6.12, 8.03)	0.38 (-1.73, 2.44)	1.29	0.1979	0.25	0.0198
***IGFBP3* Site5**	89	5.40 (4.20, 6.58)	6.23 (5.29, 7.15)	-0.21 (-2.67, 0.96)	-1.79	0.0738	0.11	0.2903
***ZNT5* Site2**	74	92.00 (81.00, 95.50)	95.50 (89.50, 97.50)	-2.50 (-12.50, 2.00)	-3.32	0.0009	0.10	0.4063
***ZNT5* Site3**	75	92.00 (82.50, 96.00)	97.00 (90.00, 99.50)	-4.00 (-12.50, 0.50)	-3.06	0.0022	0.03	0.7747

† Comparison between maternal and infant methylation levels were made by non-parametric Wilcoxon signed-rank tests. ɸ Correlations between maternal and infant methylation levels were assessed by non-parametric Spearman’s rank. * Reflects the number of paired samples with available methylation data.

The impact of maternal genotypes on infant methylation levels was explored and statistically significant findings are presented in Table 2. See Table S5 in File S1 for all results. Maternal genotypes at *DNMT1* rs2241531 and *DNMT3B* rs6058897 were associated with infant global DNA methylation levels. In both instances, the minor alleles were associated with lower DNA methylation levels. The minor alleles for the *DNMT3B* putative functional SNPs (rs1569686, rs2424913) and pairwise tagging SNPs rs992472 and rs2424923 were associated with increased *IGFBP3* methylation levels at CpG sites 1 and 5 in the infant. In particular the associations involving rs1569686, rs2424913 and rs2424928 with methylation levels at CpG site 5 were equally significant at p = 0.003, p = 0.002 and p = 0.001, respectively. However, given the large number of tests performed (10 SNPs x 13 methylation measures = 130 tests, not accounting for the 3 individual subgroups) none of these associations remain significant at the conservative Bonferroni corrected significance threshold of p = 3x10^-4^ (i.e. 0.05/130). Similarly, these associations are no longer significant following the less conservative Benjamini & Hochberg false discovery correction (corrected p-value = 0.13 for each of the three SNPs).

## Discussion

The determinants of DNA methylation patterns are the focus of considerable research interest due to their potential role in normal development and in pathogenesis [33]. Increasing evidence suggests that a wide range of environmental factors can modulate DNA methylation patterns throughout the life-course [34-39], but there is also some evidence for the involvement of heritable components in shaping the methylome [11,27,40,41]. Since the effects of heritable factors on inter-individual variation in DNA methylation patterns remain largely unknown, we investigated the impact of genetic variation in *DNMT* genes responsible for maintenance (*DNMT1*) and *de novo* methylation (*DNMT3B*) on both global and locus-specific DNA methylation in mothers and their newborn infants.

The most consistent observation in this study was the association of both maternal and infant *DNMT3B* genotype with *IGFBP3* methylation levels in the infant. In particular, the functional promoter SNP rs2424913 demonstrated associations in both instances. Equally significant were the second putative functional variant rs1569686 and 2 pairwise tagging SNPs (rs992472, rs2424923), between which strong LD was exhibited. Rs2424913 has previously been implicated in altered *DNMT3B* promoter activity and lung cancer risk [16]. Specifically, the minor allele was associated with increased promoter activity, which the authors postulated may lead to aberrant hypermethylation leading to disease risk. Subsequent studies have demonstrated association between *DNMT3B* promoter polymorphisms and hypermethylation of DNA repair and tumour suppressor genes [19,25]. However, these studies tested proxies for the rs2424913 polymorphism. Although the current findings of increased *IGFBP3* methylation with carriage of the minor rs2424913 variant are consistent with the hypothesis, they did not reach statistical significance following correction for multiple testing. Furthermore, the effect of this SNP on *IGFBP3* methylation is relatively modest and could reflect technical variability within the Pyrosequencing assay.

It was anticipated that genetic variation within the *DNMT* genes would have a more wide spread effect upon global, compared to loci-specific, DNA methylation. Two potential associations were demonstrated between maternal genotype (*DNMT1* rs2241531, *DNMT3B* rs6058897) and infant global methylation. However, these findings were not robust to correction for multiple testing.

The presence of both maternal and infant samples is a particular strength of the current study as it would have allowed for comparisons to be drawn between the effects of heritable factors upon DNA methylation at different time points. In the absence of robust associations however, we can consider differences in the methylation distributions between the maternal-infant pairs. In keeping with the hypothesis that environmental and lifestyle influences upon DNA methylation increase over time, global DNA methylation did appear more variable in the maternal compared to infant samples in the study cohort. However, for reasons that are unclear, the LUMA global methylation assays were less successful in the maternal samples compared to the infant samples. Substantial efforts were taken to ensure a high quality of DNA with triplicate digests for each sample. Furthermore, inspection of the data identified no significant differences between individuals with and without successful global methylation measures in terms of *DNMT* genotypes and demographic characteristics (i.e. maternal age, smoking status, folate titre and social economic status) (data not shown), suggesting no systematic bias had occurred. Nonetheless, further investigations may be warranted utilising an alternative measure of global DNA methylation.

In conclusion, the observations from this study provide limited evidence to suggest that common variants in the *DNMT1* and *DNMT3B* genes influence inter-individual variation in global DNA methylation and the three loci investigated here. Further work could include a more comprehensive investigation of genome-wide genetic and epigenetic factors as this study was limited by taking a targeted approach to investigating both elements.

## Supporting Information

File S1 **Tables S1-S5.** Table S1. Primer sequences, PCR and Pyrosequencing conditions. Table S2. Cohort characteristics and comparison between subgroups. Table S3. Association analysis between maternal genetic predictors and maternal methylation. Table S4. Association analysis between infants’ genetic predictors and infants’ methylation. Table S5. Association analysis between maternal genetic predictors and infants’ methylation.
(DOCX)
