# Supplementary material for: Influence of DNMT Genotype on Global and Site Specific DNA Methylation Patterns in Neonates and Pregnant Women
Source: PLoS One. 2013 Oct 1;8(10):e76506. doi: 10.1371/journal.pone.0076506 (PMC3788139; doi:10.1371/journal.pone.0076506)
Supplement: File S1 — Tables S1-S5. Table S1. Primer sequences, PCR and Pyrosequencing conditions. Table S2. Cohort characteristics and comparison between subgroups. Table S3. Association analysis between maternal genetic predictors and maternal methylation. Table S4. Association analysis between infants’ genetic predictors and infants’ methylation. Table S5. Association analysis between maternal genetic predictors and infants’ methylation. (DOCX) [file pone.0076506.s001.docx]

**Supplementary Data for Potter *et al*, Influence of DNMT genotype on global and site specific DNA methylation patterns in neonates and pregnant women**

**Table S1.** Primer sequences, PCR and Pyrosequencing conditions

|  |  |  | **PCR^ǂ^** |  |  |  |  | **Pyrosequencing^†^** |
| --- | --- | --- | --- | --- | --- | --- | --- | --- |
| **Gene** | **Forward Primer** | **Reverse Primer** | **Primer Concentration (pmol)** | **Size (bp)** | **Annealing (°C)** | **Magnesium (mM)** | **Q solution (Qiagen) (µl)** | **Sequencing Primer** |
| *IGF2** | tgg ata gga gat tga gga gaa a | Biotin-aaa ccc caa caa aaa cca ct | 5 | __ | 60 | 2.5 | 2.5 | ttt ttt agg aag tat agt ta |
| *IGFBP3* | gga att aaa ttt tag aaa g | Biotin-tct aca aaa acc aaa ata t | 10 | 232 | 40 | - | 2.5 | gag ttg tat gtt agt ttt tt |
| *ZNT5* | gga gta gga gag aag gtt atg | tca ctc ccc cat aac aaa aac | 10 | 1078 | 53 | - | - | *N/A* |
| *ZNT5 Nested***^ϕ^** | gtt tgt tga gga ggt aaa | Biotin- ctc cta acc tca aat aat c | For = 10 Rev = 8.3 | 342 | 41 | - | - | ttt ggt tgg ggg ag |

^ǂ^25µl PCR reactions were performed with 0.2µg of bisulfite treated DNA using 12.5µl Hot Star Taq mastermix (Qiagen).

^†^Assays were assessed for amplification bias and reliability as described previously (McKay *et al* 2012^28^, White *et al* 2006^29^). Despite of the low annealing temperatures of these assays, the PCR products were deemed specific following gel electrophoresis and Pyrosequencing analysis. Zero and 100% *in vitro* methylated controls were run routinely alongside samples as internal controls.

*Primer design taken from Heijmans *et al* 2007^40^

^ϕ^A nested PCR was carried out using 4µl of a larger amplified region of ZNT5.

**Table S2. Cohort characteristics and comparison between subgroups.** *There was some degree of missing data in the clinical records. **^†^**Statistical comparisons were made between those individuals comprising the mother-infant pairs and those of the remaining cohort to determine how representative the smaller subgroup was. Kruskal-Wallis test for association was performed for continuous variables; Fisher’s exact test was performed for categorical variables.

|  | Complete Group  (Mothers=333, Infants=454) | | Mother-Infant pairs  (Total=137) | | Remaining Individuals  (Mothers=196, Infants=317) | | Comparison^†^ | |
| --- | --- | --- | --- | --- | --- | --- | --- | --- |
| Characteristic | **N*** | **Median (25%, 75%)** | **N*** | **Median (25%, 75%)** | **N*** | **Median (25%, 75%)** | **Test Statistic** | ***p*** |
| Comparison of maternal characteristics |  |  |  |  |  |  |  |  |
| Mothers age, years | 308 | 28.5 (23.6, 32.5) | 128 | 28.6 (24.5, 32.4) | 180 | 28.5 (23.6, 32.6) | 0.17 | 0.678 |
| Smoked during pregnancy, number (%) | 34/155 (22%) | - | 17/72 (24%) | - | 17/83 (20%) | - | 0.22 | 0.699 |
| Comparison of infant characteristics |  |  |  |  |  |  |  |  |
| Mothers age, years | 344 | 28.6 (23.6, 32.6) | 128 | 28.6 (24.5, 32.4) | 216 | 28.6 (23.5, 32.7) | 0.04 | 0.847 |
| Smoked during pregnancy, number (%) | 51/223 (23%) | - | 17/72 (24%) | - | 34/151 (23%) | - | 0.03 | 0.866 |
| Males, number (%) | 232/446 (52%) | - | 69/132 (52%) | - | 163/314 (52%) | - | 0.00 | 1.000 |
| Gestation, weeks | 444 | 40 (39, 40) | 131 | 40 (38, 40) | 313 | 40 (39, 40) | 1.04 | 0.309 |
| Birth weight (kg) | 445 | 3.5 (3.1, 3.8) | 131 | 3.5 (3.1, 3.7) | 314 | 3.5 (3.1, 3.8) | 0.01 | 0.939 |

**Table S3. Association analysis between maternal genetic predictors and maternal methylation.** **^†^**Unless otherwise stated, association between methylation and SNP genotypes was tested under an additive model using a non-parametric test for trend. **^ɸ^**SNPs rs2241531, rs437302 and rs406193 were tested under a dominant model (with respect to the minor allele) due to their low MAF.

|  |  | **AA** | | | **Aa** | | | **aa** | | | **Additive^†^** | | **Dominant** | |
| --- | --- | --- | --- | --- | --- | --- | --- | --- | --- | --- | --- | --- | --- | --- |
| **SNPs** | **Methylation Site** | **N** | **Median**  **Methylation** | **25, 50 Percentiles** | **N** | **Median**  **Methylation** | **25, 50 Percentiles** | **N** | **Median**  **Methylation** | **25, 50 Percentiles** | **Z** | ***p*** | **Chi^2^** | ***p*** |
| rs2290684 | Global | 57 | 0.31 | 0.26, 0.41 | 104 | 0.33 | 0.28, 0.52 | 47 | 0.31 | 0.27, 0.46 | 0.88 | 0.377 |  |  |
| rs2241531**^ɸ^** |  | 186 | 0.32 | 0.27, 0.43 | 22 | 0.34 | 0.28, 0.58 | 1 | 0.23 | - | - | - | 0.15 | 0.697 |
| rs6119954 |  | 148 | 0.32 | 0.27, 0.43 | 55 | 0.32 | 0.28, 0.48 | 9 | 0.33 | 0.31, 0.41 | 0.30 | 0.766 |  |  |
| rs1569686 |  | 71 | 0.33 | 0.27, 0.43 | 96 | 0.31 | 0.27, 0.48 | 42 | 0.31 | 0.26, 0.41 | -0.85 | 0.394 |  |  |
| rs2424913 |  | 69 | 0.33 | 0.28, 0.43 | 95 | 0.31 | 0.26, 0.47 | 48 | 0.32 | 0.27, 0.45 | -0.92 | 0.359 |  |  |
| rs992472 |  | 78 | 0.33 | 0.27, 0.43 | 96 | 0.31 | 0.27, 0.46 | 38 | 0.31 | 0.26, 0.41 | -1.19 | 0.233 |  |  |
| rs2424928 |  | 66 | 0.33 | 0.27, 0.43 | 93 | 0.31 | 0.27, 0.47 | 47 | 0.31 | 0.26, 0.48 | -0.81 | 0.418 |  |  |
| rs2424932 |  | 71 | 0.32 | 0.26, 0.41 | 102 | 0.32 | 0.27, 0.49 | 39 | 0.33 | 0.27, 0.47 | 1.02 | 0.306 |  |  |
| rs6058897 |  | 58 | 0.31 | 0.27, 0.41 | 104 | 0.32 | 0.27, 0.47 | 48 | 0.34 | 0.28, 0.44 | 0.78 | 0.434 |  |  |
| rs437302**^ɸ^** |  | 165 | 0.33 | 0.27, 0.47 | 45 | 0.30 | 0.27, 0.34 | 3 | 0.44 | 0.26, 0.60 | - | - | 2.21 | 0.137 |
| rs406193**^ɸ^** |  | 167 | 0.31 | 0.27, 0.44 | 43 | 0.35 | 0.26, 0.43 | 2 | 0.51 | 0.36, 0.65 | - | - | 0.10 | 0.747 |
| rs2290684 | IGF2 Site 1 | 85 | 41.69 | 38.38, 44.32 | 152 | 42.59 | 39.41, 45.48 | 65 | 42.85 | 38.21, 46.07 | 1.79 | 0.074 |  |  |
| rs2241531**^ɸ^** |  | 270 | 42.40 | 38.41, 45.32 | 32 | 42.45 | 39.93, 45.29 | 1 | 42.51 | - | - | - | 0.42 | 0.516 |
| rs6119954 |  | 208 | 42.60 | 38.88, 45.48 | 87 | 42.26 | 38.57, 44.91 | 14 | 43.18 | 38.95, 46.33 | -0.29 | 0.774 |  |  |
| rs1569686 |  | 109 | 42.60 | 38.21, 45.57 | 139 | 42.48 | 38.57, 44.92 | 57 | 42.40 | 40.04, 45.87 | 0.51 | 0.608 |  |  |
| rs2424913 |  | 97 | 42.60 | 38.34, 45.58 | 148 | 42.30 | 38.36, 44.76 | 64 | 42.33 | 39.45, 45.74 | 0.03 | 0.973 |  |  |
| rs992472 |  | 117 | 42.57 | 38.19, 45.57 | 141 | 42.44 | 38.44, 44.66 | 52 | 42.33 | 40.03, 45.96 | 0.53 | 0.598 |  |  |
| rs2424928 |  | 94 | 42.62 | 39.10, 45.58 | 145 | 42.27 | 38.27, 44.69 | 63 | 42.26 | 39.29, 45.62 | -0.13 | 0.900 |  |  |
| rs2424932 |  | 107 | 42.40 | 38.96, 45.55 | 145 | 42.44 | 38.57, 45.00 | 55 | 42.60 | 38.21, 45.24 | -0.45 | 0.655 |  |  |
| rs6058897 |  | 82 | 42.34 | 39.61, 45.32 | 151 | 42.44 | 38.36, 45.44 | 76 | 42.62 | 38.20, 45.28 | -0.08 | 0.934 |  |  |
| rs437302**^ɸ^** |  | 246 | 42.54 | 38.63, 45.44 | 60 | 42.38 | 38.69, 45.11 | 3 | 38.08 | 37.78, 46.31 | - | - | 0.27 | 0.607 |
| rs406193**^ɸ^** |  | 235 | 42.27 | 38.41, 45.04 | 69 | 43.11 | 40.04, 45.66 | 4 | 42.73 | 27.93, 44.99 | - | - | 0.92 | 0.338 |
| rs2290684 | IGF2 Site 2 | 83 | 49.06 | 45.40, 52.62 | 154 | 50.00 | 46.50, 53.09 | 65 | 49.80 | 46.71, 52.62 | 0.90 | 0.369 |  |  |
| rs2241531**^ɸ^** |  | 270 | 49.48 | 46.19, 52.68 | 31 | 51.02 | 48.52, 53.41 | 1 | 44.94 | - | - | - | 2.31 | 0.129 |
| rs6119954 |  | 209 | 49.53 | 46.35, 53.17 | 84 | 49.99 | 46.69, 52.63 | 14 | 48.95 | 45.12, 53.08 | -0.43 | 0.667 |  |  |
| rs1569686 |  | 107 | 49.90 | 46.48, 53.90 | 136 | 49.42 | 46.36, 52.24 | 58 | 50.16 | 46.34, 53.08 | -0.25 | 0.804 |  |  |
| rs2424913 |  | 99 | 49.83 | 45.32, 53.35 | 144 | 49.28 | 46.36, 52.39 | 65 | 50.07 | 46.34, 52.94 | <0.01 | 0.998 |  |  |
| rs992472 |  | 118 | 49.66 | 45.57, 53.35 | 137 | 49.09 | 46.35, 52.35 | 53 | 50.25 | 47.44, 53.31 | 0.19 | 0.848 |  |  |
| rs2424928 |  | 96 | 49.66 | 45.45, 53.25 | 141 | 49.24 | 46.37, 52.35 | 64 | 50.16 | 46.19, 52.97 | 0.21 | 0.837 |  |  |
| rs2424932 |  | 110 | 50.23 | 46.34, 53.24 | 143 | 49.09 | 46.25, 52.13 | 52 | 49.91 | 46.75, 53.77 | -0.52 | 0.601 |  |  |
| rs6058897 |  | 81 | 50.25 | 46.34, 52.64 | 150 | 49.42 | 46.50, 52.88 | 76 | 49.34 | 45.45, 53.34 | 0.05 | 0.960 |  |  |
| rs437302**^ɸ^** |  | 246 | 49.79 | 46.34, 53.00 | 57 | 49.09 | 46.48, 52.43 | 4 | 45.89 | 44.17, 48.49 | - | - | 0.36 | 0.551 |
| rs406193**^ɸ^** |  | 235 | 49.63 | 46.03, 52.88 | 66 | 49.86 | 46.71, 53.24 | 5 | 48.63 | 39.31, 49.83 | - | - | 0.16 | 0.691 |
| rs2290684 | IGF2 Site 3 | 81 | 46.25 | 43.87, 48.81 | 150 | 47.46 | 44.59, 49.59 | 64 | 48.07 | 44.37, 50.73 | 2.23 | 0.026 |  |  |
| rs2241531**^ɸ^** |  | 264 | 47.21 | 44.25, 49.86 | 31 | 47.35 | 45.92, 49.79 | 1 | 46.40 | - | - | - | 0.16 | 0.688 |
| rs6119954 |  | 202 | 47.25 | 44.18, 49.94 | 87 | 47.47 | 44.75, 49.40 | 13 | 46.84 | 45.89, 50.48 | 0.50 | 0.619 |  |  |
| rs1569686 |  | 101 | 47.38 | 44.47, 49.82 | 138 | 47.00 | 44.22, 50.37 | 58 | 47.73 | 45.53, 49.22 | 0.14 | 0.892 |  |  |
| rs2424913 |  | 91 | 47.44 | 44.47, 49.94 | 146 | 46.73 | 43.88, 49.94 | 65 | 47.72 | 45.53, 49.40 | -0.03 | 0.975 |  |  |
| rs992472 |  | 111 | 47.30 | 44.22, 49.94 | 139 | 46.96 | 43.88, 50.03 | 53 | 47.73 | 45.53, 49.22 | 0.17 | 0.863 |  |  |
| rs2424928 |  | 88 | 47.41 | 44.43, 49.86 | 143 | 46.66 | 43.87, 49.94 | 64 | 47.73 | 45.48, 49.40 | 0.16 | 0.873 |  |  |
| rs2424932 |  | 107 | 47.64 | 44.45, 49.77 | 142 | 47.12 | 44.27, 49.94 | 51 | 47.00 | 44.83, 49.51 | -0.44 | 0.663 |  |  |
| rs6058897 |  | 83 | 47.64 | 45.00, 49.40 | 147 | 47.05 | 44.27, 50.37 | 71 | 47.30 | 44.47, 49.94 | 0.02 | 0.988 |  |  |
| rs437302**^ɸ^** |  | 245 | 47.35 | 44.34, 50.02 | 54 | 47.09 | 44.38, 49.64 | 3 | 45.26 | 43.62, 47.12 | - | - | 0.22 | 0.642 |
| rs406193**^ɸ^** |  | 230 | 47.33 | 44.38, 49.64 | 66 | 46.98 | 44.34, 50.15 | 5 | 45.58 | 40.17, 46.91 | - | - | 0.37 | 0.543 |
| rs2290684 | IGF2 Mean | 88 | 45.63 | 42.88, 47.91 | 161 | 46.94 | 43.33, 48.97 | 68 | 46.73 | 43.09, 49.44 | 1.72 | 0.086 |  |  |
| rs2241531**^ɸ^** |  | 283 | 46.36 | 42.99, 49.05 | 34 | 47.05 | 45.41, 48.80 | 1 | 44.62 | - | - | - | 0.99 | 0.320 |
| rs6119954 |  | 221 | 46.24 | 43.31, 49.05 | 89 | 46.49 | 43.34, 48.54 | 14 | 47.27 | 42.68, 49.42 | -0.10 | 0.923 |  |  |
| rs1569686 |  | 114 | 46.82 | 42.99, 49.14 | 145 | 46.22 | 43.34, 48.91 | 59 | 47.04 | 43.83, 49.03 | 0.35 | 0.729 |  |  |
| rs2424913 |  | 104 | 46.82 | 42.89, 49.21 | 154 | 46.18 | 43.31, 48.79 | 66 | 47.03 | 43.65, 49.03 | 0.17 | 0.868 |  |  |
| rs992472 |  | 125 | 46.45 | 42.71, 49.14 | 146 | 46.13 | 43.33, 48.80 | 54 | 47.05 | 43.83, 49.03 | 0.47 | 0.642 |  |  |
| rs2424928 |  | 101 | 46.74 | 42.99, 49.14 | 151 | 46.16 | 43.28, 48.54 | 65 | 47.02 | 43.65, 49.03 | 0.30 | 0.766 |  |  |
| rs2424932 |  | 112 | 47.04 | 43.46, 49.00 | 151 | 46.11 | 43.33, 49.22 | 59 | 45.59 | 43.01, 48.76 | -1.12 | 0.262 |  |  |
| rs6058897 |  | 84 | 46.55 | 43.62, 48.61 | 158 | 46.33 | 43.33, 48.91 | 81 | 46.74 | 42.70, 49.22 | -0.10 | 0.919 |  |  |
| rs437302**^ɸ^** |  | 258 | 46.48 | 43.28, 49.05 | 62 | 45.86 | 43.34, 48.90 | 4 | 44.88 | 42.18, 47.60 | - | - | 0.36 | 0.549 |
| rs406193**^ɸ^** |  | 245 | 46.54 | 43.19, 48.90 | 73 | 46.09 | 43.75, 49.28 | 5 | 45.79 | 39.74, 46.45 | - | - | 0.06 | 0.804 |
| rs2290684 | IGFBP3 Site1 | 63 | 4.84 | 3.30, 6.44 | 118 | 5.59 | 4.10, 7.01 | 57 | 5.82 | 4.53, 6.95 | 1.66 | 0.098 |  |  |
| rs2241531**^ɸ^** |  | 212 | 5.61 | 4.03, 6.90 | 26 | 4.42 | 3.29, 6.72 | 1 | 8.19 | - | - | - | 0.74 | 0.391 |
| rs6119954 |  | 167 | 5.62 | 4.02, 6.94 | 65 | 5.61 | 3.93, 6.91 | 10 | 4.68 | 3.88, 5.56 | -1.33 | 0.184 |  |  |
| rs1569686 |  | 82 | 4.88 | 3.74, 6.52 | 114 | 5.95 | 3.95, 6.98 | 41 | 5.20 | 4.12, 6.91 | 0.81 | 0.415 |  |  |
| rs2424913 |  | 79 | 5.27 | 3.70, 6.87 | 117 | 5.91 | 4.23, 6.94 | 47 | 5.20 | 3.88, 6.91 | 0.22 | 0.829 |  |  |
| rs992472 |  | 92 | 5.22 | 3.72, 6.73 | 113 | 6.01 | 4.16, 6.98 | 38 | 5.18 | 4.12, 6.91 | 0.62 | 0.513 |  |  |
| rs2424928 |  | 77 | 5.27 | 3.70, 6.87 | 114 | 5.86 | 4.23, 6.93 | 46 | 5.21 | 3.88, 6.91 | 0.30 | 0.768 |  |  |
| rs2424932 |  | 79 | 5.54 | 3.95, 7.05 | 119 | 5.71 | 3.93, 6.90 | 44 | 5.05 | 3.90, 6.70 | -0.59 | 0.554 |  |  |
| rs6058897 |  | 61 | 5.22 | 3.99, 6.90 | 118 | 5.80 | 4.02, 6.95 | 62 | 5.41 | 3.83, 6.87 | -0.16 | 0.871 |  |  |
| rs437302**^ɸ^** |  | 186 | 5.55 | 3.84, 6.98 | 53 | 5.62 | 4.28, 6.53 | 4 | 4.29 | 3.90, 5.62 | - | - | 0.01 | 0.918 |
| rs406193**^ɸ^** |  | 183 | 5.54 | 3.99, 6.91 | 56 | 5.80 | 3.72, 6.87 | 3 | 1.76 | 1.63, 8.65 | - | - | 0.01 | 0.925 |
| rs2290684 | IGFBP3 Site2 | 63 | 6.33 | 5.09, 7.21 | 118 | 6.07 | 5.14, 7.59 | 57 | 6.32 | 5.73, 7.53 | 0.58 | 0.563 |  |  |
| rs2241531**^ɸ^** |  | 212 | 6.21 | 5.18, 7.42 | 26 | 6.79 | 4.99, 8.32 | 1 | 6.24 | - | - | - | 0.56 | 0.454 |
| rs6119954 |  | 167 | 6.33 | 5.51, 7.73 | 65 | 6.15 | 4.95, 7.36 | 10 | 5.53 | 4.18, 6.08 | -2.05 | 0.041 |  |  |
| rs1569686 |  | 82 | 6.13 | 5.09, 7.21 | 114 | 6.51 | 5.33, 7.96 | 41 | 6.08 | 5.12, 7.28 | 0.71 | 0.476 |  |  |
| rs2424913 |  | 79 | 6.14 | 5.08, 7.34 | 117 | 6.45 | 5.51, 7.83 | 47 | 6.05 | 4.95, 7.57 | 0.25 | 0.800 |  |  |
| rs992472 |  | 92 | 6.19 | 5.12, 7.35 | 113 | 6.39 | 5.35, 7.83 | 38 | 6.07 | 5.12, 7.57 | 0.57 | 0.572 |  |  |
| rs2424928 |  | 77 | 6.14 | 5.09, 7.33 | 114 | 6.38 | 5.35, 7.77 | 46 | 6.07 | 5.06, 7.57 | 0.40 | 0.688 |  |  |
| rs2424932 |  | 79 | 6.19 | 5.22, 7.99 | 119 | 6.27 | 5.16, 7.41 | 44 | 6.31 | 5.28, 7.34 | -0.30 | 0.765 |  |  |
| rs6058897 |  | 61 | 6.05 | 5.30, 7.28 | 118 | 6.33 | 5.18, 7.83 | 62 | 6.19 | 5.17, 7.35 | 0.03 | 0.977 |  |  |
| rs437302**^ɸ^** |  | 186 | 6.21 | 5.14, 7.54 | 53 | 6.28 | 5.32, 7.59 | 4 | 5.55 | 5.05, 6.62 | - | - | 0.19 | 0.661 |
| rs406193**^ɸ^** |  | 183 | 6.16 | 5.14, 7.60 | 56 | 6.42 | 5.56, 7.30 | 3 | 3.21 | 2.68, 9.76 | - | - | 0.19 | 0.663 |
| rs2290684 | IGFBP3 Site3 | 63 | 5.00 | 4.06, 5.97 | 118 | 4.84 | 4.02, 6.11 | 57 | 5.24 | 4.40, 6.28 | 1.21 | 0.227 |  |  |
| rs2241531**^ɸ^** |  | 212 | 4.95 | 4.17, 6.12 | 26 | 5.01 | 3.95, 6.24 | 1 | 6.86 | - | - | - | 0.17 | 0.683 |
| rs6119954 |  | 167 | 5.00 | 4.28, 6.26 | 65 | 5.07 | 4.00, 6.00 | 10 | 4.54 | 3.51, 5.08 | -1.85 | 0.064 |  |  |
| rs1569686 |  | 82 | 4.81 | 4.02, 5.77 | 114 | 5.24 | 4.33, 6.21 | 41 | 4.83 | 3.90, 6.23 | 0.52 | 0.603 |  |  |
| rs2424913 |  | 79 | 4.83 | 3.88, 6.16 | 117 | 5.23 | 4.33, 6.14 | 47 | 4.88 | 3.84, 6.25 | 0.15 | 0.882 |  |  |
| rs992472 |  | 92 | 4.84 | 4.04, 6.17 | 113 | 5.23 | 4.36, 6.15 | 38 | 4.80 | 3.90, 5.96 | 0.08 | 0.933 |  |  |
| rs2424928 |  | 77 | 4.83 | 4.02, 6.13 | 114 | 5.22 | 4.29, 6.11 | 46 | 4.89 | 3.90, 6.25 | 0.29 | 0.774 |  |  |
| rs2424932 |  | 79 | 4.90 | 3.94, 6.23 | 119 | 5.15 | 4.37, 6.14 | 44 | 4.78 | 3.68, 6.12 | -0.20 | 0.843 |  |  |
| rs6058897 |  | 61 | 4.88 | 4.28, 6.05 | 118 | 5.22 | 4.13, 6.15 | 62 | 4.84 | 4.24, 6.16 | -0.03 | 0.979 |  |  |
| rs437302**^ɸ^** |  | 186 | 5.03 | 4.05, 6.15 | 53 | 4.93 | 4.41, 6.26 | 4 | 4.21 | 3.68, 5.81 | - | - | 0.39 | 0.530 |
| rs406193**^ɸ^** |  | 183 | 4.91 | 4.14, 6.22 | 56 | 5.22 | 4.21, 6.07 | 3 | 2.90 | 2.31, 8.61 | - | - | 0.10 | 0.753 |
| rs2290684 | IGFBP3 Site4 | 62 | 8.08 | 6.15, 9.25 | 117 | 8.26 | 7.07, 9.54 | 57 | 8.47 | 7.36, 9.60 | 1.38 | 0.168 |  |  |
| rs2241531**^ɸ^** |  | 210 | 8.23 | 6.93, 9.48 | 26 | 8.39 | 7.22, 9.74 | 1 | 10.31 | - | - | - | 0.29 | 0.591 |
| rs6119954 |  | 165 | 8.35 | 7.28, 9.54 | 65 | 8.22 | 6.57, 9.51 | 10 | 7.64 | 6.07, 9.07 | -1.49 | 0.137 |  |  |
| rs1569686 |  | 80 | 8.03 | 6.56, 9.18 | 114 | 8.55 | 7.42, 9.72 | 41 | 8.09 | 7.10, 9.91 | 1.41 | 0.158 |  |  |
| rs2424913 |  | 77 | 8.11 | 6.69, 9.38 | 117 | 8.44 | 7.44, 9.49 | 47 | 8.09 | 6.15, 9.91 | 0.69 | 0.491 |  |  |
| rs992472 |  | 90 | 8.09 | 6.58, 9.36 | 113 | 8.56 | 7.47, 9.80 | 38 | 8.02 | 7.10, 9.60 | 1.29 | 0.196 |  |  |
| rs2424928 |  | 75 | 8.11 | 6.66, 9.38 | 114 | 8.42 | 7.22, 9.48 | 46 | 8.17 | 6.63, 9.91 | 0.82 | 0.412 |  |  |
| rs2424932 |  | 79 | 8.53 | 7.27, 10.10 | 119 | 8.30 | 7.04, 9.47 | 42 | 8.11 | 6.46, 9.24 | -1.34 | 0.182 |  |  |
| rs6058897 |  | 61 | 8.31 | 7.10, 9.54 | 117 | 8.24 | 7.04, 9.48 | 61 | 8.19 | 7.08, 9.21 | -0.58 | 0.560 |  |  |
| rs437302**^ɸ^** |  | 185 | 8.31 | 7.04, 9.60 | 52 | 8.24 | 7.21, 9.39 | 4 | 6.89 | 5.20, 7.42 | - | - | 0.89 | 0.345 |
| rs406193**^ɸ^** |  | 182 | 8.25 | 7.04, 9.54 | 55 | 8.43 | 7.25, 9.49 | 3 | 4.68 | 2.72, 11.10 | - | - | <0.01 | 0.956 |
| rs2290684 | IGFBP3 Site5 | 62 | 5.91 | 4.92, 6.97 | 118 | 6.20 | 5.02, 7.45 | 57 | 6.46 | 5.02, 7.82 | 1.20 | 0.228 |  |  |
| rs2241531**^ɸ^** |  | 211 | 6.14 | 4.96, 7.51 | 26 | 6.41 | 5.28, 8.11 | 1 | 6.84 | - | - | - | 1.32 | 0.251 |
| rs6119954 |  | 167 | 6.43 | 5.18, 7.68 | 64 | 5.81 | 4.82, 7.05 | 10 | 4.96 | 4.27, 6.46 | -2.72 | 0.007 |  |  |
| rs1569686 |  | 82 | 6.15 | 5.02, 6.98 | 113 | 6.22 | 5.10, 7.75 | 41 | 6.17 | 4.83, 7.17 | -0.06 | 0.949 |  |  |
| rs2424913 |  | 79 | 6.17 | 5.02, 7.20 | 116 | 6.29 | 5.09, 7.74 | 47 | 6.06 | 4.81, 7.24 | -0.46 | 0.643 |  |  |
| rs992472 |  | 92 | 6.28 | 5.01, 7.25 | 112 | 6.18 | 5.12, 7.74 | 38 | 6.12 | 4.81, 7.24 | -0.39 | 0.699 |  |  |
| rs2424928 |  | 77 | 6.17 | 5.02, 7.14 | 113 | 6.22 | 5.08, 7.71 | 46 | 6.12 | 4.83, 7.24 | -0.27 | 0.786 |  |  |
| rs2424932 |  | 79 | 6.09 | 4.83, 7.21 | 118 | 6.38 | 5.18, 7.87 | 44 | 6.08 | 5.05, 7.42 | 0.86 | 0.387 |  |  |
| rs6058897 |  | 61 | 6.22 | 5.03, 7.27 | 117 | 6.09 | 4.91, 7.70 | 62 | 6.28 | 5.48, 7.22 | 0.47 | 0.639 |  |  |
| rs437302**^ɸ^** |  | 186 | 6.17 | 4.96, 7.51 | 52 | 6.40 | 5.06, 7.61 | 4 | 5.66 | 3.83, 6.64 | - | - | 0.29 | 0.592 |
| rs406193**^ɸ^** |  | 182 | 6.27 | 4.96, 7.54 | 56 | 6.03 | 5.23, 7.52 | 3 | 5.49 | 3.87, 11.98 | - | - | <0.01 | 0.994 |
| rs2290684 | IGFBP3 Mean | 63 | 6.08 | 4.85, 7.24 | 118 | 6.21 | 5.08, 7.46 | 57 | 6.53 | 5.55, 7.57 | 1.36 | 0.175 |  |  |
| rs2241531**^ɸ^** |  | 212 | 6.23 | 5.11, 7.32 | 26 | 6.29 | 5.12, 7.51 | 1 | 7.69 | - | **-** | - | 0.23 | 0.630 |
| rs6119954 |  | 167 | 6.32 | 5.36, 7.46 | 65 | 6.20 | 4.50, 7.36 | 10 | 5.55 | 4.48, 6.53 | -2.04 | 0.041 |  |  |
| rs1569686 |  | 82 | 6.03 | 5.02, 7.05 | 114 | 6.50 | 5.36, 7.70 | 41 | 6.07 | 5.08, 7.46 | 0.86 | 0.390 |  |  |
| rs2424913 |  | 79 | 6.14 | 5.01, 7.24 | 117 | 6.42 | 5.42, 7.51 | 47 | 6.07 | 4.60, 7.46 | 0.27 | 0.790 |  |  |
| rs992472 |  | 92 | 6.17 | 5.02, 7.22 | 113 | 6.49 | 5.42, 7.51 | 38 | 5.95 | 5.08, 7.46 | 0.58 | 0.561 |  |  |
| rs2424928 |  | 77 | 6.14 | 5.02, 7.21 | 114 | 6.38 | 5.36, 7.36 | 46 | 6.16 | 5.01, 7.46 | 0.40 | 0.688 |  |  |
| rs2424932 |  | 79 | 6.30 | 5.11, 7.68 | 119 | 6.30 | 5.30, 7.42 | 44 | 6.10 | 5.01, 7.06 | -0.42 | 0.677 |  |  |
| rs6058897 |  | 61 | 6.25 | 5.12, 7.34 | 118 | 6.23 | 5.06, 7.36 | 62 | 6.28 | 5.14, 7.24 | -0.05 | 0.962 |  |  |
| rs437302**^ɸ^** |  | 186 | 6.27 | 5.11, 7.42 | 53 | 6.24 | 5.31, 7.27 | 4 | 5.04 | 5.01, 5.74 | - | - | <0.01 | 0.969 |
| rs406193**^ɸ^** |  | 183 | 6.24 | 5.09,7.42 | 56 | 6.41 | 5.39,7.30 | 3 | 3.15 | 3.10, 10.02 | - | - | 0.08 | 0.780 |
| rs2290684 | ZNT5 Site2 | 60 | 90.75 | 81.75, 95.50 | 104 | 87.25 | 77.00, 94.50 | 47 | 92.00 | 85.00, 95.00 | 0.47 | 0.637 |  |  |
| rs2241531**^ɸ^** |  | 186 | 90.25 | 80.00, 95.00 | 23 | 88.50 | 77.00, 96.00 | 0 | - | - | - | - | 0.13 | 0.719 |
| rs6119954 |  | 140 | 90.50 | 77.25, 95.00 | 66 | 89.75 | 81.50, 94.00 | 9 | 95.50 | 93.50, 96.00 | 0.79 | 0.430 |  |  |
| rs1569686 |  | 82 | 92.00 | 81.00, 96.00 | 92 | 89.75 | 78.25, 94.00 | 38 | 90.75 | 82.50, 95.50 | -0.49 | 0.627 |  |  |
| rs2424913 |  | 71 | 92.00 | 77.50, 96.00 | 102 | 90.00 | 79.50, 94.00 | 41 | 91.50 | 83.00, 94.50 | -0.22 | 0.826 |  |  |
| rs992472 |  | 85 | 92.00 | 81.00, 95.50 | 94 | 89.75 | 77.50, 94.00 | 35 | 91.00 | 82.50, 95.50 | -0.68 | 0.495 |  |  |
| rs2424928 |  | 68 | 92.00 | 77.25, 96.00 | 102 | 89.75 | 79.00, 94.00 | 41 | 91.50 | 83.00, 94.50 | -0.08 | 0.933 |  |  |
| rs2424932 |  | 79 | 90.00 | 82.50, 95.00 | 94 | 90.75 | 77.50, 94.50 | 40 | 89.50 | 77.25, 96.00 | 0.15 | 0.880 |  |  |
| rs6058897 |  | 51 | 90.50 | 79.00, 94.50 | 108 | 90.00 | 79.50, 94.50 | 55 | 92.00 | 77.50, 96.00 | 0.77 | 0.441 |  |  |
| rs437302**^ɸ^** |  | 172 | 90.75 | 79.25, 95.00 | 39 | 88.50 | 79.50, 94.50 | 3 | 96.00 | 93.00, 99.00 | - | - | 0.12 | 0.730 |
| rs406193**^ɸ^** |  | 160 | 90.00 | 81.00, 94.50 | 50 | 91.75 | 76.00, 95.50 | 2 | 85.75 | 75.50, 96.00 | - | - | 0.25 | 0.621 |
| rs2290684 | ZNT5 Site3 | 59 | 91.00 | 81.50, 94.00 | 102 | 93.50 | 86.50, 96.50 | 48 | 93.50 | 88.00, 95.75 | 2.05 | 0.041 |  |  |
| rs2241531**^ɸ^** |  | 184 | 91.75 | 84.75, 95.50 | 22 | 93.75 | 87.00, 95.50 | 0 | - | - | - | - | 0.65 | 0.421 |
| rs6119954 |  | 137 | 92.50 | 84.00, 95.50 | 65 | 92.00 | 86.00, 95.50 | 10 | 91.25 | 83.00, 97.00 | 0.90 | 0.368 |  |  |
| rs1569686 |  | 80 | 91.50 | 84.50, 95.25 | 90 | 92.50 | 85.00, 95.50 | 39 | 91.50 | 86.00, 96.50 | 1.21 | 0.228 |  |  |
| rs2424913 |  | 69 | 91.00 | 84.00, 95.00 | 100 | 92.75 | 85.25, 95.50 | 42 | 91.50 | 86.00, 96.50 | 1.23 | 0.219 |  |  |
| rs992472 |  | 83 | 92.00 | 84.00, 95.50 | 92 | 93.00 | 85.25, 95.50 | 36 | 91.50 | 85.00, 96.50 | 1.03 | 0.304 |  |  |
| rs2424928 |  | 67 | 91.00 | 84.00, 95.00 | 100 | 92.75 | 85.25, 95.50 | 42 | 91.50 | 86.00, 96.50 | 1.22 | 0.223 |  |  |
| rs2424932 |  | 81 | 91.50 | 85.00, 96.50 | 90 | 92.50 | 86.50, 95.50 | 39 | 92.00 | 82.00, 95.50 | -0.97 | 0.330 |  |  |
| rs6058897 |  | 51 | 91.50 | 83.00, 96.50 | 105 | 93.00 | 86.50, 96.00 | 55 | 89.50 | 82.50, 95.00 | -1.33 | 0.183 |  |  |
| rs437302**^ɸ^** |  | 170 | 92.50 | 85.00, 95.50 | 39 | 91.00 | 84.00, 95.00 | 3 | 96.00 | 93.50, 96.50 | - | - | 0.56 | 0.456 |
| rs406193**^ɸ^** |  | 158 | 92.25 | 86.50, 95.50 | 49 | 91.00 | 80.50, 95.50 | 2 | 94.50 | 93.50, 95.50 | - | - | 1.11 | 0.291 |
|  | ZNT5 Site4 | dropped |  |  |  |  |  |  |  |  |  |  |  |  |
|  | ZNT5 Site5 | dropped |  |  |  |  |  |  |  |  |  |  |  |  |
|  | ZNT5 Site6 | dropped |  |  |  |  |  |  |  |  |  |  |  |  |

**Table S4**. **Association analysis between infants’ genetic predictors and infants’ methylation.** **^†^**Unless otherwise stated, association between methylation and SNP genotypes was tested under an additive model using a non-parametric test for trend. **^ɸ^**SNPs rs2241531, rs437302 and rs406193 were tested under a dominant model (with respect to the minor allele) due to their low MAF.

|  |  | **AA** | | | **Aa** | | | **aa** | | | **Additive^†^** | | **Dominant** | |
| --- | --- | --- | --- | --- | --- | --- | --- | --- | --- | --- | --- | --- | --- | --- |
| **SNPs** | **Methylation Site** | **N** | **Median**  **Methylation** | **25, 50 Percentiles** | **N** | **Median**  **Methylation** | **25, 50 Percentiles** | **N** | **Median**  **Methylation** | **25, 50 Percentiles** | **Z** | ***p*** | **Chi^2^** | ***p*** |
| rs2290684 | Global | 90 | 0.36 | 0.32, 0.41 | 153 | 0.36 | 0.30, 0.40 | 69 | 0.37 | 0.32, 0.42 | 0.65 | 0.513 |  |  |
| rs2241531**^ɸ^** |  | 281 | 0.36 | 0.31, 0.41 | 37 | 0.37 | 0.31, 0.41 | 1 | 0.35 | - | - | - | 0.22 | 0.643 |
| rs6119954 |  | 209 | 0.37 | 0.32, 0.42 | 103 | 0.34 | 0.30, 0.40 | 6 | 0.32 | 0.30, 0.34 | -2.98 | 0.003 |  |  |
| rs1569686 |  | 110 | 0.36 | 0.32, 0.42 | 155 | 0.36 | 0.32, 0.40 | 52 | 0.34 | 0.30, 0.41 | -1.19 | 0.232 |  |  |
| rs2424913 |  | 96 | 0.36 | 0.32, 0.42 | 156 | 0.37 | 0.32, 0.41 | 67 | 0.34 | 0.30, 0.42 | -0.65 | 0.516 |  |  |
| rs992472 |  | 115 | 0.36 | 0.32, 0.42 | 160 | 0.36 | 0.32, 0.40 | 45 | 0.34 | 0.30, 0.42 | -0.83 | 0.409 |  |  |
| rs2424928 |  | 94 | 0.36 | 0.32, 0.42 | 153 | 0.37 | 0.32, 0.41 | 66 | 0.34 | 0.30, 0.42 | -0.66 | 0.509 |  |  |
| rs2424932 |  | 112 | 0.35 | 0.30, 0.40 | 155 | 0.37 | 0.33, 0.41 | 48 | 0.34 | 0.30, 0.42 | 0.66 | 0.506 |  |  |
| rs6058897 |  | 87 | 0.34 | 0.30, 0.40 | 158 | 0.36 | 0.32, 0.40 | 71 | 0.37 | 0.34, 0.42 | 1.69 | 0.091 |  |  |
| rs437302**^ɸ^** |  | 261 | 0.36 | 0.31, 0.41 | 52 | 0.36 | 0.31, 0.40 | 3 | 0.35 | 0.28, 0.39 | - | - | 0.31 | 0.577 |
| rs406193**^ɸ^** |  | 240 | 0.36 | 0.31, 0.40 | 70 | 0.37 | 0.31, 0.42 | 5 | 0.42 | 0.34, 0.43 | - | - | 0.08 | 0.780 |
| rs2290684 | IGF2 Site 1 | 100 | 46.05 | 42.52, 48.51 | 206 | 45.38 | 41.28, 48.13 | 91 | 45.10 | 42.15, 47.78 | -0.85 | 0.396 |  |  |
| rs2241531**^ɸ^** |  | 355 | 45.36 | 41.85, 48.18 | 47 | 45.35 | 41.60, 47.47 | 1 | 49.67 | - | - | - | 0.06 | 0.815 |
| rs6119954 |  | 254 | 45.36 | 41.63, 47.89 | 142 | 45.54 | 42.21, 48.55 | 5 | 40.56 | 39.40, 42.77 | 0.01 | 0.991 |  |  |
| rs1569686 |  | 130 | 45.71 | 42.10, 48.28 | 199 | 45.35 | 41.50, 47.49 | 73 | 45.13 | 41.95, 48.22 | -0.33 | 0.739 |  |  |
| rs2424913 |  | 113 | 45.54 | 42.37, 48.15 | 201 | 45.54 | 41.63, 48.21 | 90 | 45.04 | 41.85, 47.70 | -0.58 | 0.564 |  |  |
| rs992472 |  | 139 | 45.41 | 41.69, 48.18 | 203 | 45.35 | 41.50, 47.47 | 63 | 46.06 | 42.07, 49.32 | 0.52 | 0.600 |  |  |
| rs2424928 |  | 109 | 45.72 | 42.37, 48.18 | 201 | 45.35 | 41.60, 47.56 | 88 | 45.12 | 41.90, 47.93 | -0.60 | 0.550 |  |  |
| rs2424932 |  | 143 | 45.24 | 41.70, 48.47 | 190 | 45.47 | 41.89, 48.01 | 67 | 45.41 | 41.63, 48.18 | -0.21 | 0.831 |  |  |
| rs6058897 |  | 119 | 45.13 | 41.97, 48.25 | 195 | 45.54 | 41.50, 48.01 | 84 | 45.55 | 42.58, 48.17 | 0.34 | 0.735 |  |  |
| rs437302**^ɸ^** |  | 324 | 45.28 | 41.67, 47.80 | 73 | 45.90 | 42.65, 48.21 | 4 | 46.27 | 43.37, 47.69 | - | - | 0.31 | 0.580 |
| rs406193**^ɸ^** |  | 307 | 45.52 | 41.69, 48.15 | 86 | 45.45 | 42.78, 48.28 | 8 | 44.59 | 41.62, 45.57 | - | - | 0.01 | 0.911 |
| rs2290684 | IGF2 Site 2 | 98 | 52.14 | 49.78, 55.46 | 206 | 51.81 | 49.34, 55.05 | 91 | 51.88 | 49.97, 53.89 | -1.14 | 0.256 |  |  |
| rs2241531**^ɸ^** |  | 353 | 51.84 | 49.56, 54.77 | 47 | 52.57 | 49.34, 55.44 | 1 | 55.77 | - |  |  | 1.43 | 0.232 |
| rs6119954 |  | 260 | 51.73 | 49.64, 54.75 | 134 | 52.15 | 49.71, 55.02 | 5 | 48.34 | 47.80, 48.50 | -0.23 | 0.820 |  |  |
| rs1569686 |  | 134 | 52.05 | 49.80, 54.73 | 199 | 51.88 | 49.55, 54.78 | 67 | 51.89 | 49.10, 55.56 | -0.03 | 0.977 |  |  |
| rs2424913 |  | 116 | 52.13 | 50.17, 54.67 | 202 | 51.81 | 49.39, 54.78 | 84 | 52.09 | 49.07, 55.54 | <0.01 | 1.000 |  |  |
| rs992472 |  | 144 | 51.84 | 49.70, 54.67 | 202 | 51.88 | 49.55, 54.52 | 57 | 52.07 | 49.55, 56.19 | 0.47 | 0.636 |  |  |
| rs2424928 |  | 112 | 52.52 | 50.17, 54.73 | 201 | 51.76 | 49.36, 54.78 | 83 | 52.11 | 49.10, 55.56 | <0.01 | 0.999 |  |  |
| rs2424932 |  | 139 | 51.88 | 49.03, 54.73 | 191 | 51.88 | 49.97, 55.02 | 68 | 53.00 | 49.74, 54.92 | 0.71 | 0.478 |  |  |
| rs6058897 |  | 112 | 52.09 | 49.58, 55.54 | 199 | 51.78 | 49.28, 54.92 | 86 | 52.66 | 50.10, 54.60 | -0.07 | 0.944 |  |  |
| rs437302**^ɸ^** |  | 325 | 51.88 | 49.55, 54.73 | 70 | 51.95 | 48.62, 54.92 | 4 | 53.11 | 51.53, 54.88 | - | - | <0.01 | 0.951 |
| rs406193**^ɸ^** |  | 303 | 51.88 | 49.36, 54.78 | 88 | 52.36 | 50.00, 54.92 | 8 | 50.61 | 49.75, 52.56 | - | - | 0.52 | 0.473 |
| rs2290684 | IGF2 Site 3 | 103 | 50.94 | 48.06, 53.06 | 205 | 49.75 | 46.86, 52.16 | 93 | 50.28 | 48.52, 52.27 | -0.72 | 0.473 |  |  |
| rs2241531**^ɸ^** |  | 356 | 50.12 | 47.20, 52.29 | 50 | 49.96 | 47.19, 52.27 | 1 | 55.42 | - | - | - | <0.01 | 0.983 |
| rs6119954 |  | 260 | 50.23 | 47.42, 52.22 | 142 | 50.10 | 47.17, 52.82 | 4 | 47.17 | 45.95, 51.13 | -0.17 | 0.863 |  |  |
| rs1569686 |  | 137 | 50.31 | 47.52, 52.08 | 200 | 49.85 | 47.14, 52.17 | 70 | 50.53 | 47.34, 53.06 | 0.52 | 0.606 |  |  |
| rs2424913 |  | 117 | 50.37 | 47.62, 52.07 | 204 | 49.85 | 47.15, 52.29 | 88 | 50.49 | 46.93, 52.73 | 0.29 | 0.772 |  |  |
| rs992472 |  | 146 | 50.00 | 47.52, 52.08 | 202 | 49.97 | 47.11, 52.18 | 61 | 50.58 | 47.35, 53.27 | 0.87 | 0.386 |  |  |
| rs2424928 |  | 114 | 50.51 | 47.67, 52.08 | 202 | 49.79 | 47.11, 52.21 | 86 | 50.52 | 46.99, 52.82 | 0.18 | 0.858 |  |  |
| rs2424932 |  | 139 | 50.47 | 46.86, 52.84 | 196 | 49.97 | 47.53, 52.17 | 70 | 49.63 | 47.14, 52.07 | -0.61 | 0.545 |  |  |
| rs6058897 |  | 118 | 50.20 | 47.35, 52.82 | 196 | 49.93 | 47.08, 52.29 | 88 | 50.66 | 47.85, 52.16 | -0.17 | 0.864 |  |  |
| rs437302**^ɸ^** |  | 330 | 50.02 | 47.18, 52.27 | 71 | 50.37 | 47.52, 52.80 | 4 | 50.71 | 48.96, 52.17 | - | - | 0.09 | 0.759 |
| rs406193**^ɸ^** |  | 306 | 50.12 | 47.16, 52.36 | 91 | 50.44 | 47.36, 52.74 | 8 | 48.72 | 48.12, 48.87 | - | - | <0.01 | 0.961 |
| rs2290684 | IGF2 Mean | 105 | 49.63 | 46.95, 51.80 | 215 | 48.99 | 45.70, 51.55 | 95 | 49.36 | 47.04, 51.09 | -0.96 | 0.339 |  |  |
| rs2241531**^ɸ^** |  | 370 | 49.19 | 46.47, 51.55 | 50 | 49.79 | 46.09, 51.49 | 1 | 53.62 | - | - | - | 0.16 | 0.689 |
| rs6119954 |  | 270 | 49.36 | 46.49, 51.54 | 144 | 49.37 | 46.42, 51.79 | 5 | 45.36 | 44.38, 45.58 | -0.40 | 0.690 |  |  |
| rs1569686 |  | 139 | 49.50 | 47.02, 51.79 | 208 | 48.98 | 46.28, 51.44 | 73 | 49.56 | 45.92, 51.55 | -0.28 | 0.781 |  |  |
| rs2424913 |  | 120 | 49.47 | 47.02, 51.72 | 211 | 49.15 | 46.26, 51.66 | 91 | 49.38 | 45.92, 51.40 | -0.29 | 0.768 |  |  |
| rs992472 |  | 149 | 49.37 | 46.75, 51.65 | 211 | 49.01 | 46.26, 51.39 | 63 | 50.05 | 45.99, 52.73 | 0.35 | 0.729 |  |  |
| rs2424928 |  | 116 | 49.63 | 47.02, 51.79 | 210 | 48.93 | 46.24, 51.54 | 89 | 49.56 | 45.99, 51.40 | -0.42 | 0.674 |  |  |
| rs2424932 |  | 147 | 49.30 | 46.09, 51.55 | 200 | 49.14 | 46.72, 51.55 | 71 | 49.37 | 47.02, 51.79 | 0.23 | 0.816 |  |  |
| rs6058897 |  | 122 | 49.24 | 46.29, 51.45 | 205 | 49.24 | 46.24, 51.57 | 89 | 49.83 | 47.20, 51.80 | 0.48 | 0.634 |  |  |
| rs437302**^ɸ^** |  | 341 | 49.19 | 46.31, 51.49 | 74 | 49.36 | 46.84, 51.43 | 4 | 50.12 | 48.54, 50.99 | - | - | 0.08 | 0.783 |
| rs406193**^ɸ^** |  | 320 | 49.33 | 46.28, 51.47 | 91 | 49.71 | 47.16, 52.12 | 8 | 47.91 | 47.27, 48.55 | - | - | 0.52 | 0.472 |
| rs2290684 | IGFBP3 Site1 | 79 | 4.92 | 3.77, 5.91 | 166 | 4.68 | 3.88, 5.46 | 70 | 5.25 | 4.50, 6.24 | 1.45 | 0.147 |  |  |
| rs2241531**^ɸ^** |  | 282 | 4.83 | 4.09, 5.70 | 38 | 5.04 | 3.71, 5.93 | 0 | - | - | - | - | <0.01 | 0.954 |
| rs6119954 |  | 201 | 4.83 | 4.09, 5.70 | 114 | 4.87 | 3.95, 6.16 | 4 | 5.03 | 4.55, 6.63 | 0.56 | 0.573 |  |  |
| rs1569686 |  | 105 | 4.83 | 3.74, 5.70 | 161 | 4.89 | 4.11, 5.68 | 55 | 4.81 | 3.88, 6.18 | 0.60 | 0.546 |  |  |
| rs2424913 |  | 90 | 4.79 | 3.52, 5.70 | 163 | 4.91 | 4.11, 5.68 | 68 | 4.87 | 4.08, 6.31 | 1.22 | 0.221 |  |  |
| rs992472 |  | 114 | 4.81 | 3.52, 5.68 | 161 | 4.88 | 4.17, 5.77 | 47 | 4.92 | 4.04, 6.18 | 1.19 | 0.236 |  |  |
| rs2424928 |  | 86 | 4.79 | 3.71, 5.70 | 165 | 4.92 | 4.09, 5.75 | 66 | 4.83 | 4.06, 6.18 | 0.89 | 0.375 |  |  |
| rs2424932 |  | 118 | 5.02 | 4.03, 6.18 | 141 | 4.73 | 4.11, 5.54 | 61 | 4.79 | 3.74, 5.75 | -1.15 | 0.248 |  |  |
| rs6058897 |  | 87 | 4.85 | 4.13, 5.96 | 163 | 4.92 | 3.88, 5.84 | 65 | 4.79 | 3.71, 5.64 | -1.04 | 0.299 |  |  |
| rs437302**^ɸ^** |  | 258 | 4.90 | 4.09, 5.68 | 58 | 4.67 | 3.47, 5.91 | 3 | 10.31 | 3.74, 14.81 | - | - | 0.34 | 0.558 |
| rs406193**^ɸ^** |  | 248 | 4.84 | 3.93, 5.78 | 66 | 4.89 | 4.17, 5.55 | 7 | 4.84 | 3.46, 5.39 | - | - | 0.06 | 0.807 |
| rs2290684 | IGFBP3 Site2 | 80 | 5.92 | 5.12, 6.63 | 167 | 5.98 | 5.37, 6.68 | 69 | 5.95 | 5.44, 6.99 | 1.15 | 0.249 |  |  |
| rs2241531**^ɸ^** |  | 282 | 5.95 | 5.37, 6.70 | 39 | 6.17 | 4.87, 6.80 | 0 | - | - | - | - | <0.01 | 0.958 |
| rs6119954 |  | 205 | 5.96 | 5.32, 6.68 | 111 | 5.94 | 5.30, 6.93 | 4 | 6.42 | 5.08, 7.61 | 0.25 | 0.802 |  |  |
| rs1569686 |  | 107 | 5.96 | 5.24, 6.55 | 160 | 5.90 | 5.40, 6.95 | 55 | 6.19 | 5.24, 6.84 | 0.73 | 0.464 |  |  |
| rs2424913 |  | 92 | 5.93 | 5.23, 6.56 | 162 | 5.91 | 5.38, 6.70 | 68 | 6.20 | 5.27, 7.12 | 1.15 | 0.251 |  |  |
| rs992472 |  | 116 | 5.97 | 5.25, 6.55 | 160 | 5.89 | 5.34, 6.98 | 47 | 6.28 | 5.44, 6.84 | 0.87 | 0.386 |  |  |
| rs2424928 |  | 88 | 5.94 | 5.25, 6.56 | 164 | 5.90 | 5.37, 6.86 | 66 | 6.19 | 5.24, 6.84 | 0.73 | 0.463 |  |  |
| rs2424932 |  | 117 | 6.15 | 5.17, 7.06 | 143 | 5.90 | 5.37, 6.63 | 61 | 5.97 | 5.32, 6.55 | -0.79 | 0.432 |  |  |
| rs6058897 |  | 87 | 6.18 | 5.34, 7.06 | 163 | 5.90 | 5.33, 6.72 | 66 | 5.95 | 5.30, 6.55 | -0.88 | 0.377 |  |  |
| rs437302**^ɸ^** |  | 258 | 5.93 | 5.33, 6.73 | 59 | 6.03 | 5.14, 6.63 | 3 | 10.47 | 5.94, 15.71 | - | - | 0.05 | 0.822 |
| rs406193**^ɸ^** |  | 249 | 5.99 | 5.24, 6.84 | 66 | 5.84 | 5.47, 6.44 | 7 | 6.09 | 5.96, 6.79 | - | - | 0.95 | 0.329 |
| rs2290684 | IGFBP3 Site3 | 79 | 4.26 | 3.74, 5.03 | 167 | 4.39 | 3.89, 4.94 | 69 | 4.66 | 4.09, 5.64 | 2.45 | 0.014 |  |  |
| rs2241531**^ɸ^** |  | 282 | 4.43 | 3.94, 5.18 | 38 | 4.48 | 3.92, 5.20 | 0 | - | - | - | - | 0.01 | 0.912 |
| rs6119954 |  | 202 | 4.43 | 3.89, 5.03 | 113 | 4.49 | 4.05, 5.37 | 4 | 4.55 | 4.06, 5.95 | 0.83 | 0.404 |  |  |
| rs1569686 |  | 105 | 4.30 | 3.76, 4.94 | 163 | 4.50 | 4.01, 5.37 | 53 | 4.43 | 3.94, 5.15 | 1.68 | 0.093 |  |  |
| rs2424913 |  | 90 | 4.29 | 3.74, 4.85 | 165 | 4.49 | 4.00, 5.29 | 66 | 4.53 | 4.03, 5.67 | 2.50 | 0.012 |  |  |
| rs992472 |  | 114 | 4.30 | 3.76, 4.88 | 163 | 4.50 | 3.94, 5.41 | 45 | 4.51 | 4.09, 5.15 | 2.23 | 0.026 |  |  |
| rs2424928 |  | 86 | 4.30 | 3.75, 4.85 | 167 | 4.49 | 3.97, 5.29 | 65 | 4.51 | 4.03, 5.67 | 2.16 | 0.030 |  |  |
| rs2424932 |  | 118 | 4.50 | 4.03, 5.60 | 141 | 4.41 | 3.89, 5.03 | 61 | 4.38 | 3.74, 4.88 | -1.81 | 0.071 |  |  |
| rs6058897 |  | 85 | 4.54 | 4.01, 5.29 | 166 | 4.49 | 3.97, 5.32 | 64 | 4.29 | 3.81, 4.60 | -2.07 | 0.039 |  |  |
| rs437302**^ɸ^** |  | 258 | 4.42 | 3.94, 5.17 | 58 | 4.45 | 3.78, 5.01 | 3 | 6.54 | 4.38, 14.32 | - | - | 0.05 | 0.830 |
| rs406193**^ɸ^** |  | 248 | 4.50 | 3.99, 5.33 | 66 | 4.29 | 3.78, 4.85 | 7 | 4.30 | 3.74, 4.54 | - | - | 0.53 | 0.021 |
| rs2290684 | IGFBP3 Site4 | 78 | 7.23 | 6.60, 8.33 | 169 | 7.42 | 6.46, 8.17 | 70 | 7.69 | 6.68, 8.41 | 1.06 | 0.288 |  |  |
| rs2241531**^ɸ^** |  | 284 | 7.45 | 6.60, 8.24 | 38 | 7.11 | 6.20, 8.35 | 0 | - | - | - | - | 0.65 | 0.420 |
| rs6119954 |  | 204 | 7.45 | 6.60, 8.17 | 113 | 7.27 | 6.41, 8.30 | 4 | 8.22 | 7.55, 9.15 | 0.08 | 0.934 |  |  |
| rs1569686 |  | 106 | 7.24 | 6.46, 7.88 | 163 | 7.44 | 6.61, 8.50 | 54 | 7.57 | 6.61, 8.46 | 1.48 | 0.139 |  |  |
| rs2424913 |  | 91 | 7.20 | 6.40, 7.88 | 165 | 7.43 | 6.61, 8.27 | 67 | 7.64 | 6.61, 8.62 | 1.85 | 0.064 |  |  |
| rs992472 |  | 115 | 7.29 | 6.46, 7.90 | 163 | 7.41 | 6.52, 8.50 | 46 | 7.66 | 6.74, 8.47 | 1.74 | 0.082 |  |  |
| rs2424928 |  | 87 | 7.23 | 6.46, 7.88 | 167 | 7.41 | 6.52, 8.36 | 65 | 7.5 | 6.61, 8.46 | 1.45 | 0.147 |  |  |
| rs2424932 |  | 119 | 7.48 | 6.40, 8.47 | 143 | 7.41 | 6.51, 8.27 | 60 | 7.34 | 6.66, 7.84 | -0.61 | 0.540 |  |  |
| rs6058897 |  | 86 | 7.56 | 6.61, 8.39 | 165 | 7.46 | 6.62, 8.27 | 66 | 7.08 | 6.46, 7.87 | -1.64 | 0.102 |  |  |
| rs437302**^ɸ^** |  | 259 | 7.36 | 6.46, 8.21 | 59 | 7.48 | 6.70, 8.27 | 3 | 11.54 | 7.60, 18.77 | - | - | 1.27 | 0.259 |
| rs406193**^ɸ^** |  | 250 | 7.48 | 6.41, 8.35 | 66 | 7.23 | 6.65, 7.70 | 7 | 6.87 | 6.66, 7.76 | - | - | 1.45 | 0.229 |
| rs2290684 | IGFBP3 Site5 | 79 | 6.32 | 5.40, 7.45 | 165 | 6.63 | 5.73, 7.56 | 68 | 6.90 | 5.97, 8.05 | 2.09 | 0.036 |  |  |
| rs2241531**^ɸ^** |  | 280 | 6.59 | 5.74, 7.69 | 37 | 5.75 | 4.18, 7.15 | 0 | - | - | - | - | 5.00 | 0.025 |
| rs6119954 |  | 202 | 6.54 | 5.73, 7.56 | 110 | 6.72 | 5.45, 7.65 | 4 | 7.67 | 4.89, 8.50 | 0.26 | 0.793 |  |  |
| rs1569686 |  | 107 | 6.32 | 5.61, 7.24 | 158 | 6.68 | 5.58, 7.68 | 53 | 6.83 | 5.96, 8.12 | 2.03 | 0.042 |  |  |
| rs2424913 |  | 92 | 6.24 | 5.49, 7.22 | 160 | 6.57 | 5.62, 7.64 | 66 | 6.83 | 5.77, 8.15 | 2.23 | 0.026 |  |  |
| rs992472 |  | 115 | 6.32 | 5.61, 7.24 | 158 | 6.65 | 5.58, 7.68 | 46 | 7.02 | 6.07, 8.18 | 2.33 | 0.020 |  |  |
| rs2424928 |  | 88 | 6.24 | 5.58, 7.22 | 162 | 6.57 | 5.62, 7.65 | 64 | 6.83 | 5.75, 8.11 | 1.88 | 0.060 |  |  |
| rs2424932 |  | 116 | 6.80 | 5.78, 8.11 | 140 | 6.39 | 5.58, 7.42 | 61 | 6.51 | 5.72, 7.45 | -1.23 | 0.217 |  |  |
| rs6058897 |  | 85 | 6.79 | 5.77, 8.12 | 161 | 6.55 | 5.62, 7.55 | 66 | 6.20 | 5.42, 7.26 | -2.05 | 0.040 |  |  |
| rs437302**^ɸ^** |  | 254 | 6.57 | 5.72, 7.56 | 59 | 6.51 | 5.38, 7.58 | 3 | 13.55 | 5.42, 16.88 | - | - | 0.12 | 0.733 |
| rs406193**^ɸ^** |  | 246 | 6.60 | 5.65, 7.80 | 65 | 6.36 | 5.57, 7.19 | 7 | 6.37 | 5.98, 9.54 | - | - | 1.14 | 0.286 |
| rs2290684 | IGFBP3 Mean | 80 | 5.78 | 5.29, 6.50 | 169 | 5.75 | 5.17, 6.49 | 70 | 6.02 | 5.53, 6.93 | 1.76 | 0.079 |  |  |
| rs2241531**^ɸ^** |  | 285 | 5.85 | 5.24, 6.59 | 39 | 5.72 | 4.72, 6.77 | 0 | - | - | **-** | - | 0.85 | 0.355 |
| rs6119954 |  | 205 | 5.82 | 5.29, 6.53 | 114 | 5.86 | 5.10, 6.77 | 4 | 6.38 | 5.35, 7.44 | 0.33 | 0.745 |  |  |
| rs1569686 |  | 107 | 5.71 | 5.17, 6.45 | 163 | 5.85 | 5.28, 6.80 | 55 | 5.93 | 5.20, 7.11 | 1.39 | 0.163 |  |  |
| rs2424913 |  | 92 | 5.64 | 5.11, 6.42 | 165 | 5.85 | 5.36, 6.62 | 68 | 5.95 | 5.21, 7.04 | 1.92 | 0.055 |  |  |
| rs992472 |  | 116 | 5.69 | 5.17, 6.35 | 163 | 5.85 | 5.24, 6.83 | 47 | 5.99 | 5.24, 7.11 | 1.92 | 0.054 |  |  |
| rs2424928 |  | 88 | 5.68 | 5.15, 6.42 | 167 | 5.85 | 5.36, 6.68 | 66 | 5.91 | 5.20, 6.96 | 1.51 | 0.130 |  |  |
| rs2424932 |  | 120 | 5.93 | 5.22, 7.16 | 143 | 5.82 | 5.24, 6.56 | 61 | 5.77 | 5.32, 6.45 | -1.17 | 0.241 |  |  |
| rs6058897 |  | 87 | 5.90 | 5.24, 6.95 | 166 | 5.86 | 5.28, 6.58 | 66 | 5.58 | 5.17, 6.39 | -1.75 | 0.080 |  |  |
| rs437302**^ɸ^** |  | 261 | 5.83 | 5.24, 6.59 | 59 | 5.83 | 5.10, 6.54 | 3 | 10.48 | 5.42, 16.10 | - | - | <0.01 | 1.000 |
| rs406193**^ɸ^** |  | 252 | 5.86 | 5.23, 6.79 | 66 | 5.76 | 5.32, 6.09 | 7 | 5.48 | 5.34, 6.76 | - | - | 1.34 | 0.247 |
| rs2290684 | ZNT5 Site2 | 84 | 94.00 | 89.00, 96.00 | 171 | 94.00 | 86.50, 97.50 | 77 | 96.00 | 89.50, 97.50 | 1.62 | 0.106 |  |  |
| rs2241531**^ɸ^** |  | 304 | 94.25 | 88.00, 97.00 | 35 | 95.00 | 86.00, 97.50 | 1 | 95.50 | - | - | - | 0.19 | 0.664 |
| rs6119954 |  | 216 | 94.00 | 87.50, 97.00 | 115 | 95.00 | 88.00, 97.50 | 5 | 96.00 | 86.50, 97.00 | 0.62 | 0.537 |  |  |
| rs1569686 |  | 114 | 94.00 | 85.50, 97.00 | 172 | 94.25 | 88.00, 97.25 | 53 | 95.50 | 91.50, 97.00 | 1.09 | 0.274 |  |  |
| rs2424913 |  | 101 | 94.50 | 87.00, 97.00 | 172 | 94.00 | 87.75, 97.00 | 67 | 95.00 | 90.50, 97.50 | 0.67 | 0.504 |  |  |
| rs992472 |  | 122 | 94.00 | 86.00, 97.00 | 175 | 94.50 | 88.00, 97.50 | 44 | 95.00 | 91.00, 97.00 | 1.00 | 0.319 |  |  |
| rs2424928 |  | 97 | 94.50 | 87.00, 97.00 | 172 | 94.00 | 87.00, 97.00 | 66 | 95.00 | 90.50, 97.50 | 0.60 | 0.546 |  |  |
| rs2424932 |  | 119 | 95.00 | 89.50, 97.00 | 162 | 94.00 | 88.00, 97.00 | 55 | 94.00 | 84.00, 97.00 | -1.16 | 0.244 |  |  |
| rs6058897 |  | 90 | 95.50 | 90.50, 97.50 | 169 | 94.50 | 88.00, 97.00 | 76 | 93.50 | 85.75, 97.00 | -1.30 | 0.192 |  |  |
| rs437302**^ɸ^** |  | 277 | 95.00 | 88.00, 97.50 | 58 | 92.75 | 84.50, 96.00 | 3 | 95.00 | 82.00, 97.50 | - | - | 3.72 | 0.054 |
| rs406193**^ɸ^** |  | 252 | 95.00 | 88.00, 97.00 | 76 | 94.50 | 88.25, 97.50 | 8 | 92.50 | 87.25, 96.25 | - | - | 0.12 | 0.732 |
| rs2290684 | ZNT5 Site3 | 82 | 93.75 | 89.50, 99.00 | 173 | 96.50 | 88.00, 99.00 | 78 | 96.25 | 92.00, 99.50 | 1.23 | 0.217 |  |  |
| rs2241531**^ɸ^** |  | 304 | 96.50 | 89.25, 99.00 | 36 | 95.25 | 91.50, 98.25 | 1 | 80.00 | - | - | - | 0.62 | 0.431 |
| rs6119954 |  | 216 | 95.50 | 88.75, 98.50 | 117 | 96.50 | 90.50, 99.00 | 4 | 97.25 | 63.00, 98.00 | 0.66 | 0.510 |  |  |
| rs1569686 |  | 115 | 95.50 | 87.00, 98.50 | 173 | 96.50 | 90.00, 99.00 | 52 | 96.00 | 88.00, 98.25 | 0.24 | 0.813 |  |  |
| rs2424913 |  | 102 | 96.00 | 88.00, 98.50 | 173 | 96.50 | 90.50, 99.00 | 66 | 95.50 | 89.00, 98.00 | -0.47 | 0.637 |  |  |
| rs992472 |  | 123 | 95.50 | 88.00, 98.50 | 176 | 96.50 | 89.75, 99.00 | 43 | 95.50 | 84.50, 98.00 | -0.11 | 0.912 |  |  |
| rs2424928 |  | 98 | 96.00 | 88.00, 98.50 | 173 | 96.50 | 90.50, 99.00 | 65 | 95.00 | 89.00, 98.00 | -0.49 | 0.624 |  |  |
| rs2424932 |  | 119 | 96.00 | 87.50, 99.00 | 163 | 96.50 | 89.50, 99.00 | 55 | 95.50 | 84.00, 98.50 | -0.10 | 0.922 |  |  |
| rs6058897 |  | 89 | 96.00 | 89.50, 98.50 | 171 | 96.50 | 89.00, 99.00 | 76 | 95.50 | 90.25, 98.75 | 0.34 | 0.733 |  |  |
| rs437302**^ɸ^** |  | 278 | 96.25 | 89.50, 99.00 | 58 | 96.25 | 86.50, 99.00 | 3 | 81.00 | 39.00, 97.50 | - | - | 0.19 | 0.660 |
| rs406193**^ɸ^** |  | 253 | 96.50 | 89.00, 98.50 | 76 | 95.50 | 91.50, 99.25 | 8 | 97.25 | 95.25, 99.75 | - | - | 0.73 | 0.392 |
|  | ZNT5 Site4 | dropped |  |  |  |  |  |  |  |  |  |  |  |  |
|  | ZNT5 Site5 | dropped |  |  |  |  |  |  |  |  |  |  |  |  |
|  | ZNT5 Site6 | dropped |  |  |  |  |  |  |  |  |  |  |  |  |

**Table S5**. **Association analysis between maternal genetic predictors and infants’ methylation.** **^†^**Unless otherwise stated, association between methylation and SNP genotypes was tested under an additive model using a non-parametric test for trend. **^ɸ^**SNPs rs2241531, rs437302 and rs406193 were tested under a dominant model (with respect to the minor allele) due to their low MAF.

|  |  | **AA** | | | **Aa** | | | **aa** | | | **Additive^†^** | | **Dominant** | |
| --- | --- | --- | --- | --- | --- | --- | --- | --- | --- | --- | --- | --- | --- | --- |
| **SNPs** | **Methylation Site** | **N** | **Median**  **Methylation** | **25, 50**  **Percentiles** | **N** | **Median**  **Methylation** | **25, 50 Percentiles** | **N** | **Median**  **Methylation** | **25, 50 Percentiles** | **Z** | ***p*** | **Chi^2^** | ***p*** |
| rs2290684 | Global | 29 | 0.35 | 0.33, 0.41 | 49 | 0.38 | 0.32, 0.41 | 20 | 0.35 | 0.29, 0.39 | -1.05 | 0.296 |  |  |
| rs2241531**^ɸ^** |  | 81 | 0.35 | 0.30, 0.40 | 18 | 0.39 | 0.35, 0.42 | 0 | - | - | - | - | 4.35 | 0.037 |
| rs6119954 |  | 66 | 0.37 | 0.34, 0.41 | 26 | 0.35 | 0.29, 0.40 | 8 | 0.33 | 0.29, 0.39 | -1.95 | 0.051 |  |  |
| rs1569686 |  | 40 | 0.39 | 0.34, 0.41 | 40 | 0.35 | 0.30, 0.39 | 20 | 0.37 | 0.32, 0.41 | -1.35 | 0.177 |  |  |
| rs2424913 |  | 37 | 0.39 | 0.34, 0.41 | 43 | 0.35 | 0.30, 0.39 | 21 | 0.36 | 0.31, 0.40 | -1.47 | 0.143 |  |  |
| rs992472 |  | 41 | 0.39 | 0.34, 0.41 | 40 | 0.35 | 0.30, 0.38 | 19 | 0.36 | 0.31, 0.41 | -1.55 | 0.122 |  |  |
| rs2424928 |  | 36 | 0.39 | 0.34, 0.41 | 42 | 0.35 | 0.30, 0.39 | 21 | 0.36 | 0.31, 0.40 | -1.39 | 0.166 |  |  |
| rs2424932 |  | 38 | 0.37 | 0.32, 0.41 | 38 | 0.35 | 0.29, 0.39 | 25 | 0.38 | 0.35, 0.40 | 0.60 | 0.547 |  |  |
| rs6058897 |  | 27 | 0.36 | 0.30, 0.39 | 49 | 0.35 | 0.30, 0.40 | 25 | 0.39 | 0.35, 0.41 | 2.21 | 0.027 |  |  |
| rs437302**^ɸ^** |  | 78 | 0.36 | 0.32, 0.40 | 20 | 0.36 | 0.30, 0.43 | 2 | 0.37 | 0.34, 0.39 | - | - | 0.07 | 0.796 |
| rs406193**^ɸ^** |  | 79 | 0.36 | 0.31, 0.41 | 20 | 0.38 | 0.33, 0.40 | 1 | 0.37 | - | - | - | 0.32 | 0.573 |
| rs2290684 | IGF2 Site 1 | 34 | 46.16 | 41.97, 48.67 | 61 | 45.36 | 40.44, 47.18 | 26 | 45.20 | 41.85, 47.78 | -0.62 | 0.538 |  |  |
| rs2241531**^ɸ^** |  | 102 | 45.36 | 40.56, 47.32 | 20 | 46.35 | 42.74, 48.85 | 0 | - | - | - | - | 1.48 | 0.224 |
| rs6119954 |  | 81 | 45.54 | 41.22, 47.81 | 31 | 45.36 | 40.44, 47.64 | 11 | 45.54 | 42.57, 46.06 | -0.50 | 0.615 |  |  |
| rs1569686 |  | 45 | 46.50 | 42.21, 48.50 | 55 | 45.36 | 39.76, 47.20 | 22 | 45.10 | 40.97, 46.08 | -1.32 | 0.186 |  |  |
| rs2424913 |  | 42 | 46.33 | 41.60, 48.50 | 56 | 45.39 | 39.58, 47.16 | 26 | 45.20 | 41.97, 46.08 | -0.96 | 0.338 |  |  |
| rs992472 |  | 49 | 46.16 | 41.60, 48.15 | 53 | 45.36 | 39.40, 47.11 | 21 | 45.54 | 41.97, 46.08 | -1.01 | 0.310 |  |  |
| rs2424928 |  | 41 | 46.16 | 41.60, 48.50 | 55 | 45.41 | 39.40, 47.20 | 26 | 45.20 | 41.97, 46.08 | -0.85 | 0.393 |  |  |
| rs2424932 |  | 42 | 45.74 | 40.97, 47.53 | 56 | 45.00 | 41.32, 47.19 | 26 | 46.01 | 40.83, 48.50 | 0.44 | 0.657 |  |  |
| rs6058897 |  | 33 | 44.86 | 41.02, 46.48 | 59 | 45.54 | 39.76, 47.32 | 32 | 45.76 | 41.51, 48.39 | 0.80 | 0.421 |  |  |
| rs437302**^ɸ^** |  | 95 | 45.10 | 40.97, 47.20 | 26 | 46.12 | 41.22, 47.78 | 2 | 49.30 | 47.89, 50.70 | - | - | 1.45 | 0.229 |
| rs406193**^ɸ^** |  | 93 | 45.97 | 41.22, 47.81 | 28 | 45.20 | 41.90, 47.09 | 1 | 18.61 | - | - | - | 0.53 | 0.465 |
| rs2290684 | IGF2 Site 2 | 33 | 53.28 | 47.90, 54.73 | 60 | 51.37 | 48.82, 53.80 | 25 | 52.13 | 48.75, 54.24 | -0.49 | 0.627 |  |  |
| rs2241531**^ɸ^** |  | 101 | 51.86 | 48.24, 54.16 | 18 | 53.11 | 51.18, 54.73 | 0 | - | - | - | - | 1.75 | 0.186 |
| rs6119954 |  | 79 | 51.49 | 48.50, 54.16 | 30 | 52.15 | 48.13, 54.32 | 11 | 52.28 | 48.62, 55.02 | 0.73 | 0.465 |  |  |
| rs1569686 |  | 45 | 52.76 | 49.48, 54.73 | 52 | 51.53 | 48.02, 53.57 | 22 | 51.47 | 48.34, 54.05 | -1.49 | 0.136 |  |  |
| rs2424913 |  | 42 | 52.73 | 49.27, 54.73 | 54 | 51.47 | 48.13, 53.75 | 25 | 52.05 | 48.62, 53.58 | -1.21 | 0.224 |  |  |
| rs992472 |  | 49 | 52.57 | 49.27, 54.73 | 50 | 51.58 | 48.13, 53.75 | 21 | 52.05 | 48.34, 54.05 | -1.05 | 0.295 |  |  |
| rs2424928 |  | 41 | 52.69 | 49.27, 54.73 | 53 | 51.45 | 48.13, 53.75 | 25 | 52.05 | 48.62, 53.58 | -1.11 | 0.268 |  |  |
| rs2424932 |  | 41 | 51.89 | 48.23, 54.32 | 55 | 51.57 | 48.50, 53.57 | 25 | 52.76 | 49.48, 55.58 | 1.24 | 0.213 |  |  |
| rs6058897 |  | 32 | 51.60 | 48.48, 53.82 | 56 | 51.73 | 48.44, 54.28 | 33 | 52.57 | 48.75, 54.16 | 0.52 | 0.602 |  |  |
| rs437302**^ɸ^** |  | 93 | 51.45 | 48.38, 54.05 | 25 | 53.33 | 49.56, 54.21 | 2 | 53.22 | 51.84, 54.60 | - | - | 2.46 | 0.117 |
| rs406193**^ɸ^** |  | 92 | 51.72 | 48.21, 54.19 | 26 | 52.34 | 49.48, 55.19 | 1 | 23.32 | - | - | - | 1.02 | 0.313 |
| rs2290684 | IGF2 Site 3 | 34 | 49.73 | 45.33, 52.22 | 60 | 48.93 | 46.70, 51.90 | 26 | 50.66 | 48.53, 52.78 | 0.91 | 0.364 |  |  |
| rs2241531**^ɸ^** |  | 101 | 49.89 | 45.85, 52.27 | 20 | 50.03 | 48.40, 51.72 | 0 | - | - | - | - | 0.27 | 0.603 |
| rs6119954 |  | 81 | 49.64 | 46.61, 52.28 | 31 | 50.04 | 45.73, 52.07 | 10 | 50.38 | 49.30, 51.96 | 0.57 | 0.570 |  |  |
| rs1569686 |  | 46 | 50.83 | 47.18, 53.20 | 54 | 49.28 | 44.82, 51.05 | 21 | 49.42 | 47.47, 52.82 | -0.88 | 0.377 |  |  |
| rs2424913 |  | 43 | 50.75 | 47.05, 53.20 | 55 | 49.14 | 44.82, 50.94 | 25 | 49.42 | 47.63, 52.64 | -0.55 | 0.581 |  |  |
| rs992472 |  | 50 | 50.43 | 47.05, 52.97 | 52 | 49.53 | 44.59, 51.41 | 20 | 49.81 | 47.02, 53.04 | -0.51 | 0.611 |  |  |
| rs2424928 |  | 42 | 50.60 | 47.05, 53.20 | 54 | 49.28 | 44.82, 50.94 | 25 | 49.42 | 47.63, 52.64 | -0.43 | 0.665 |  |  |
| rs2424932 |  | 41 | 50.19 | 46.40, 52.28 | 56 | 49.07 | 45.65, 51.80 | 26 | 50.67 | 47.05, 53.20 | 0.61 | 0.542 |  |  |
| rs6058897 |  | 32 | 49.36 | 46.94, 52.73 | 57 | 49.89 | 46.61, 52.07 | 34 | 50.43 | 47.00, 52.90 | 0.02 | 0.981 |  |  |
| rs437302**^ɸ^** |  | 95 | 49.42 | 45.73, 52.22 | 25 | 50.44 | 48.45, 52.07 | 2 | 51.20 | 49.50, 52.90 | - | - | 1.78 | 0.182 |
| rs406193**^ɸ^** |  | 92 | 49.77 | 45.84, 52.46 | 28 | 50.09 | 47.68, 51.74 | 1 | 26.16 | - | - | - | <0.01 | 0.993 |
| rs2290684 | IGF2 Mean | 34 | 50.08 | 44.40, 51.41 | 62 | 48.54 | 45.33, 50.86 | 27 | 49.75 | 45.83, 51.34 | -0.08 | 0.937 |  |  |
| rs2241531**^ɸ^** |  | 104 | 48.93 | 45.31, 50.96 | 20 | 50.08 | 47.43, 51.40 | 0 | - | - | - | - | 1.05 | 0.305 |
| rs6119954 |  | 83 | 49.10 | 45.41, 51.28 | 31 | 49.37 | 44.38, 51.19 | 11 | 49.56 | 46.53, 50.70 | 0.17 | 0.864 |  |  |
| rs1569686 |  | 47 | 50.41 | 46.29, 51.66 | 55 | 48.72 | 44.40, 50.62 | 22 | 48.55 | 45.33, 50.99 | -1.45 | 0.147 |  |  |
| rs2424913 |  | 44 | 50.49 | 46.23, 51.58 | 56 | 48.54 | 44.84, 50.63 | 26 | 48.67 | 46.09, 50.32 | -1.20 | 0.231 |  |  |
| rs992472 |  | 51 | 49.98 | 46.17, 51.49 | 53 | 48.72 | 45.28, 50.62 | 21 | 48.80 | 45.33, 50.99 | -1.07 | 0.286 |  |  |
| rs2424928 |  | 43 | 50.41 | 46.17, 51.49 | 55 | 48.72 | 44.40, 50.63 | 26 | 48.67 | 46.09, 50.32 | -1.09 | 0.278 |  |  |
| rs2424932 |  | 42 | 49.12 | 44.40, 51.28 | 57 | 48.55 | 45.47, 50.82 | 27 | 50.06 | 45.61, 51.80 | 0.72 | 0.470 |  |  |
| rs6058897 |  | 33 | 48.55 | 45.41, 50.86 | 59 | 49.10 | 45.28, 50.93 | 34 | 50.23 | 46.17, 51.41 | 0.81 | 0.417 |  |  |
| rs437302**^ɸ^** |  | 97 | 48.72 | 45.36, 50.86 | 26 | 50.61 | 45.47, 51.25 | 2 | 51.24 | 50.68, 51.80 | - | - | 2.83 | 0.093 |
| rs406193**^ɸ^** |  | 95 | 49.26 | 45.23, 51.22 | 28 | 49.37 | 46.20, 50.94 | 1 | 22.70 | - | - | - | <0.01 | 0.979 |
| rs2290684 | IGFBP3 Site1 | 27 | 4.83 | 3.70, 5.75 | 48 | 4.60 | 2.83, 5.18 | 21 | 4.59 | 3.00, 6.12 | -0.67 | 0.504 |  |  |
| rs2241531**^ɸ^** |  | 78 | 4.69 | 3.09, 5.52 | 17 | 3.77 | 2.94, 5.04 | 0 | - | - | - | - | 0.57 | 0.449 |
| rs6119954 |  | 64 | 4.60 | 2.98, 5.38 | 23 | 4.75 | 3.09, 5.91 | 9 | 5.09 | 2.70, 5.58 | 0.62 | 0.534 |  |  |
| rs1569686 |  | 37 | 3.77 | 2.77, 5.36 | 41 | 4.61 | 3.70, 5.28 | 17 | 5.09 | 3.09, 6.41 | 1.96 | 0.050 |  |  |
| rs2424913 |  | 36 | 3.76 | 2.40, 5.20 | 40 | 4.61 | 3.65, 5.40 | 21 | 5.09 | 4.65, 6.41 | 2.34 | 0.019 |  |  |
| rs992472 |  | 40 | 4.38 | 2.79, 5.27 | 40 | 4.63 | 3.65, 5.40 | 16 | 5.23 | 3.84, 6.60 | 1.99 | 0.046 |  |  |
| rs2424928 |  | 35 | 3.74 | 2.03, 5.04 | 39 | 4.61 | 3.59, 5.28 | 21 | 5.09 | 4.65, 6.41 | 2.56 | 0.011 |  |  |
| rs2424932 |  | 30 | 4.87 | 3.09, 5.58 | 46 | 4.75 | 3.56, 5.39 | 21 | 3.74 | 1.75, 5.04 | -1.52 | 0.129 |  |  |
| rs6058897 |  | 27 | 4.92 | 4.11, 6.16 | 43 | 4.60 | 2.95, 5.52 | 27 | 4.50 | 2.80, 5.39 | -1.24 | 0.217 |  |  |
| rs437302**^ɸ^** |  | 75 | 4.65 | 3.09, 5.41 | 20 | 4.71 | 2.88, 5.56 | 2 | 7.04 | 3.77, 10.31 | - | - | <0.01 | 0.976 |
| rs406193**^ɸ^** |  | 72 | 4.78 | 3.20, 5.47 | 22 | 4.55 | 2.95, 5.78 | 1 | 2.03 | - | - | - | 0.53 | 0.468 |
| rs2290684 | IGFBP3 Site2 | 27 | 5.59 | 4.98, 6.63 | 49 | 5.66 | 5.06, 6.39 | 21 | 5.68 | 5.49, 6.21 | 0.08 | 0.938 |  |  |
| rs2241531**^ɸ^** |  | 79 | 5.68 | 5.14, 6.42 | 17 | 5.38 | 5.12, 6.52 | 0 | - | - | - | - | 0.17 | 0.676 |
| rs6119954 |  | 66 | 5.67 | 5.17, 6.42 | 23 | 5.59 | 4.86, 6.46 | 8 | 6.10 | 4.79, 7.04 | 0.39 | 0.700 |  |  |
| rs1569686 |  | 39 | 5.68 | 5.14, 6.42 | 41 | 5.55 | 5.06, 6.18 | 16 | 6.08 | 5.10, 7.98 | 0.98 | 0.328 |  |  |
| rs2424913 |  | 38 | 5.70 | 5.14, 6.44 | 40 | 5.55 | 5.02, 6.09 | 20 | 6.19 | 5.42, 7.29 | 1.24 | 0.216 |  |  |
| rs992472 |  | 42 | 5.70 | 5.15, 6.44 | 40 | 5.57 | 5.02, 6.19 | 15 | 5.98 | 4.86, 8.44 | 0.71 | 0.476 |  |  |
| rs2424928 |  | 37 | 5.68 | 5.14, 6.42 | 39 | 5.54 | 4.98, 6.09 | 20 | 6.19 | 5.42, 7.29 | 1.41 | 0.157 |  |  |
| rs2424932 |  | 29 | 5.84 | 4.78, 6.52 | 48 | 5.73 | 5.40, 6.44 | 21 | 5.49 | 4.80, 6.09 | -0.56 | 0.573 |  |  |
| rs6058897 |  | 26 | 6.04 | 5.34, 6.69 | 43 | 5.54 | 4.89, 6.09 | 29 | 5.89 | 5.24, 6.44 | -0.55 | 0.584 |  |  |
| rs437302**^ɸ^** |  | 74 | 5.67 | 5.06, 6.29 | 22 | 5.64 | 5.14, 6.44 | 2 | 8.18 | 5.89, 10.47 | - | - | 0.54 | 0.465 |
| rs406193**^ɸ^** |  | 73 | 5.74 | 5.06, 6.46 | 22 | 5.61 | 5.24, 6.09 | 1 | 3.21 | - | - | - | 0.60 | 0.440 |
| rs2290684 | IGFBP3 Site3 | 27 | 4.26 | 3.84, 5.02 | 50 | 4.34 | 3.65, 4.89 | 20 | 4.37 | 4.03, 4.86 | 0.45 | 0.656 |  |  |
| rs2241531**^ɸ^** |  | 79 | 4.35 | 3.87, 4.93 | 17 | 4.29 | 3.88, 5.02 | 0 | - | - | - | - | <0.01 | 0.962 |
| rs6119954 |  | 65 | 4.38 | 3.82, 4.94 | 23 | 4.26 | 3.62, 4.73 | 9 | 4.14 | 3.88, 4.93 | -0.49 | 0.622 |  |  |
| rs1569686 |  | 39 | 4.34 | 3.76, 4.94 | 40 | 4.24 | 3.68, 4.77 | 17 | 4.50 | 3.88, 5.93 | 0.55 | 0.580 |  |  |
| rs2424913 |  | 38 | 4.36 | 3.88, 4.94 | 39 | 4.22 | 3.65, 4.74 | 21 | 4.50 | 3.88, 5.67 | 0.47 | 0.640 |  |  |
| rs992472 |  | 42 | 4.39 | 3.88, 4.93 | 39 | 4.22 | 3.65, 4.75 | 16 | 4.62 | 3.88, 5.96 | 0.39 | 0.694 |  |  |
| rs2424928 |  | 37 | 4.34 | 3.88, 4.93 | 38 | 4.21 | 3.65, 4.73 | 21 | 4.50 | 3.88, 5.67 | 0.61 | 0.540 |  |  |
| rs2424932 |  | 30 | 4.38 | 3.84, 4.94 | 47 | 4.29 | 3.88, 4.94 | 21 | 4.38 | 3.73, 4.71 | -0.47 | 0.641 |  |  |
| rs6058897 |  | 27 | 4.35 | 3.84, 5.03 | 42 | 4.24 | 3.71, 4.74 | 29 | 4.38 | 3.92, 4.94 | 0.12 | 0.903 |  |  |
| rs437302**^ɸ^** |  | 74 | 4.33 | 3.82, 4.93 | 22 | 4.23 | 3.92, 4.89 | 2 | 6.36 | 6.17, 6.54 | - | - | 0.24 | 0.626 |
| rs406193**^ɸ^** |  | 74 | 4.35 | 3.82, 4.94 | 21 | 4.31 | 3.92, 4.65 | 1 | 0.89 | - | - | - | 0.52 | 0.469 |
| rs2290684 | IGFBP3 Site4 | 27 | 7.11 | 6.26, 7.99 | 49 | 6.83 | 6.18, 7.90 | 21 | 7.60 | 6.16, 8.04 | -0.07 | 0.945 |  |  |
| rs2241531**^ɸ^** |  | 79 | 7.22 | 6.18, 8.01 | 17 | 7.00 | 6.12, 7.58 | 0 | - | - | - | - | 0.16 | 0.687 |
| rs6119954 |  | 65 | 6.99 | 6.16, 8.01 | 23 | 7.58 | 6.26, 7.90 | 9 | 7.00 | 6.18, 7.80 | 0.70 | 0.481 |  |  |
| rs1569686 |  | 38 | 6.82 | 5.67, 8.01 | 41 | 7.11 | 6.26, 7.71 | 17 | 7.65 | 6.72, 8.30 | 1.51 | 0.130 |  |  |
| rs2424913 |  | 37 | 6.99 | 5.67, 8.01 | 40 | 7.11 | 6.22, 7.73 | 21 | 7.58 | 6.71, 8.30 | 1.47 | 0.141 |  |  |
| rs992472 |  | 41 | 6.80 | 6.07, 7.99 | 40 | 7.17 | 6.32, 7.83 | 16 | 7.67 | 6.45, 8.98 | 1.66 | 0.096 |  |  |
| rs2424928 |  | 36 | 6.91 | 5.23, 8.00 | 39 | 7.11 | 6.18, 7.71 | 21 | 7.58 | 6.71, 8.30 | 1.67 | 0.095 |  |  |
| rs2424932 |  | 30 | 7.01 | 6.07, 7.90 | 48 | 7.17 | 6.39, 8.00 | 20 | 6.90 | 2.89, 8.01 | -0.55 | 0.584 |  |  |
| rs6058897 |  | 27 | 7.50 | 6.57, 8.03 | 42 | 7.11 | 6.07, 7.90 | 29 | 6.99 | 6.12, 8.04 | -0.91 | 0.365 |  |  |
| rs437302**^ɸ^** |  | 74 | 7.01 | 6.12, 7.90 | 22 | 7.17 | 6.26, 8.35 | 2 | 10.08 | 8.61, 11.54 | - | - | 0.92 | 0.338 |
| rs406193**^ɸ^** |  | 73 | 7.11 | 6.26, 8.01 | 22 | 6.97 | 5.67, 7.70 | 1 | 2.10 | - | - | - | 0.39 | 0.531 |
| rs2290684 | IGFBP3 Site5 | 27 | 6.12 | 4.77, 7.29 | 50 | 6.22 | 4.54, 6.98 | 20 | 6.27 | 5.70, 7.49 | 0.58 | 0.559 |  |  |
| rs2241531**^ɸ^** |  | 78 | 6.22 | 5.36, 7.15 | 17 | 6.12 | 4.59, 6.55 | 0 | - | - | - | - | 0.39 | 0.534 |
| rs6119954 |  | 65 | 6.11 | 4.77, 6.88 | 22 | 6.32 | 5.29, 7.48 | 9 | 6.88 | 5.73, 8.18 | 1.57 | 0.117 |  |  |
| rs1569686 |  | 38 | 5.99 | 3.10, 6.51 | 40 | 6.27 | 4.66, 6.89 | 17 | 6.88 | 6.21, 8.19 | 2.96 | 0.003 |  |  |
| rs2424913 |  | 37 | 6.00 | 3.10, 6.88 | 39 | 6.23 | 4.54, 6.79 | 21 | 6.94 | 6.21, 8.22 | 3.05 | 0.002 |  |  |
| rs992472 |  | 41 | 6.00 | 4.48, 6.88 | 39 | 6.31 | 4.54, 7.00 | 16 | 6.80 | 6.11, 8.21 | 2.64 | 0.008 |  |  |
| rs2424928 |  | 36 | 5.99 | 2.98, 6.70 | 39 | 6.23 | 4.54, 6.79 | 21 | 6.94 | 6.21, 8.22 | 3.28 | 0.001 |  |  |
| rs2424932 |  | 30 | 6.53 | 4.59, 7.48 | 46 | 6.33 | 5.39, 6.98 | 21 | 5.64 | 2.85, 6.31 | -1.69 | 0.091 |  |  |
| rs6058897 |  | 27 | 6.67 | 5.75, 8.19 | 42 | 6.07 | 3.10, 6.79 | 28 | 6.04 | 5.35, 6.99 | -1.76 | 0.079 |  |  |
| rs437302**^ɸ^** |  | 74 | 6.20 | 4.59, 7.07 | 21 | 6.24 | 5.62, 6.98 | 2 | 9.44 | 5.33, 13.55 | - | - | 0.03 | 0.862 |
| rs406193**^ɸ^** |  | 72 | 6.23 | 5.31, 7.00 | 22 | 6.10 | 4.77, 6.83 | 1 | 1.69 | - | - | - | 1.53 | 0.216 |
| rs2290684 | IGFBP3 Mean | 27 | 5.66 | 5.24, 6.49 | 50 | 5.60 | 4.73, 6.02 | 21 | 5.75 | 5.17, 6.69 | 0.14 | 0.893 |  |  |
| rs2241531**^ɸ^** |  | 80 | 5.71 | 4.94, 6.42 | 17 | 5.52 | 5.22, 5.95 | 0 | - | - | **-** | - | 0.26 | 0.612 |
| rs6119954 |  | 66 | 5.53 | 4.91, 6.30 | 23 | 5.79 | 4.97, 6.49 | 9 | 5.85 | 4.73, 6.60 | 0.69 | 0.493 |  |  |
| rs1569686 |  | 39 | 5.42 | 4.72, 6.27 | 41 | 5.68 | 5.22, 6.02 | 17 | 5.86 | 5.24, 6.77 | 1.92 | 0.055 |  |  |
| rs2424913 |  | 38 | 5.35 | 4.72, 6.30 | 40 | 5.60 | 5.23, 5.92 | 21 | 6.02 | 5.24, 6.77 | 2.05 | 0.041 |  |  |
| rs992472 |  | 42 | 5.45 | 4.74, 6.27 | 40 | 5.71 | 5.18, 6.15 | 16 | 6.13 | 4.98, 7.37 | 1.79 | 0.074 |  |  |
| rs2424928 |  | 37 | 5.29 | 4.72, 6.27 | 39 | 5.52 | 5.22, 5.87 | 21 | 6.02 | 5.24, 6.77 | 2.26 | 0.024 |  |  |
| rs2424932 |  | 30 | 5.69 | 4.72, 6.56 | 48 | 5.71 | 5.23, 6.41 | 21 | 5.42 | 3.25, 5.95 | -1.15 | 0.249 |  |  |
| rs6058897 |  | 27 | 5.85 | 5.13, 6.60 | 43 | 5.52 | 4.97, 5.97 | 29 | 5.58 | 4.88, 6.47 | -1.01 | 0.313 |  |  |
| rs437302**^ɸ^** |  | 75 | 5.63 | 4.74, 6.36 | 22 | 5.59 | 4.97, 6.45 | 2 | 8.22 | 5.95, 10.48 | - | - | 0.26 | 0.613 |
| rs406193**^ɸ^** |  | 74 | 5.67 | 4.91, 6.45 | 22 | 5.48 | 5.17, 5.87 | 1 | 1.98 | - | - | - | 1.12 | 0.289 |
| rs2290684 | ZNT5 Site2 | 30 | 95.25 | 91.00, 96.00 | 54 | 95.50 | 89.50, 97.50 | 22 | 94.50 | 87.50, 98.00 | 0.30 | 0.767 |  |  |
| rs2241531**^ɸ^** |  | 86 | 95.50 | 90.50, 97.50 | 20 | 95.25 | 90.00, 97.50 | 0 | - | - | - | - | <0.01 | 0.994 |
| rs6119954 |  | 73 | 95.00 | 87.50, 97.50 | 25 | 95.00 | 90.50, 97.00 | 10 | 95.75 | 91.50, 98.00 | 0.84 | 0.402 |  |  |
| rs1569686 |  | 45 | 95.00 | 85.50, 97.50 | 44 | 95.00 | 90.50, 97.00 | 18 | 96.00 | 91.50, 98.00 | 1.58 | 0.113 |  |  |
| rs2424913 |  | 43 | 95.00 | 85.50, 97.50 | 45 | 95.50 | 92.50, 97.00 | 21 | 95.50 | 90.50, 98.00 | 0.91 | 0.360 |  |  |
| rs992472 |  | 49 | 95.00 | 85.50, 97.50 | 42 | 95.00 | 90.50, 97.00 | 17 | 96.00 | 95.50, 98.00 | 1.88 | 0.060 |  |  |
| rs2424928 |  | 43 | 95.00 | 85.50, 97.50 | 44 | 95.25 | 92.25, 97.00 | 21 | 95.50 | 90.50, 98.00 | 0.89 | 0.373 |  |  |
| rs2424932 |  | 38 | 95.50 | 91.00, 97.50 | 46 | 95.25 | 90.50, 97.00 | 25 | 94.00 | 82.00, 97.50 | -1.30 | 0.194 |  |  |
| rs6058897 |  | 26 | 95.50 | 90.50, 98.00 | 52 | 95.25 | 89.50, 97.25 | 31 | 95.00 | 85.50, 97.50 | -0.66 | 0.509 |  |  |
| rs437302**^ɸ^** |  | 85 | 95.50 | 90.50, 97.50 | 22 | 95.75 | 85.00, 98.00 | 1 | 75.00 | - | - | - | 0.02 | 0.880 |
| rs406193**^ɸ^** |  | 81 | 95.50 | 89.50, 97.50 | 25 | 95.50 | 92.50, 97.50 | 1 | 94.00 | - | - | - | 0.31 | 0.580 |
| rs2290684 | ZNT5 Site3 | 30 | 94.75 | 90.00, 98.00 | 55 | 97.50 | 89.50, 99.50 | 23 | 97.50 | 87.00, 99.00 | 0.90 | 0.367 |  |  |
| rs2241531**^ɸ^** |  | 88 | 97.50 | 88.75, 99.50 | 20 | 94.50 | 92.00, 98.00 | 0 | - | - | - | - | 1.11 | 0.293 |
| rs6119954 |  | 75 | 97.00 | 91.00, 99.00 | 25 | 95.50 | 87.00, 100.00 | 10 | 97.75 | 95.50, 98.50 | 0.18 | 0.855 |  |  |
| rs1569686 |  | 46 | 97.50 | 93.50, 99.50 | 45 | 93.50 | 85.50, 98.00 | 18 | 97.75 | 95.50, 99.50 | -0.36 | 0.717 |  |  |
| rs2424913 |  | 44 | 97.50 | 92.25, 99.25 | 46 | 94.50 | 86.00, 98.00 | 21 | 97.50 | 95.50, 99.50 | -0.16 | 0.874 |  |  |
| rs992472 |  | 50 | 97.25 | 90.00, 99.50 | 43 | 94.00 | 87.00, 98.00 | 17 | 98.00 | 96.00, 99.50 | 0.67 | 0.505 |  |  |
| rs2424928 |  | 44 | 97.50 | 92.25, 99.25 | 45 | 94.00 | 86.00, 98.00 | 21 | 97.50 | 95.50, 99.50 | -0.15 | 0.878 |  |  |
| rs2424932 |  | 38 | 97.00 | 93.00, 99.50 | 48 | 95.75 | 86.50, 98.00 | 25 | 97.50 | 93.50, 99.50 | -0.33 | 0.741 |  |  |
| rs6058897 |  | 27 | 97.00 | 91.00, 99.00 | 52 | 95.50 | 85.75, 99.50 | 32 | 97.25 | 93.50, 99.00 | 0.36 | 0.720 |  |  |
| rs437302**^ɸ^** |  | 87 | 97.00 | 91.00, 99.50 | 22 | 95.50 | 86.00, 98.00 | 1 | 98.50 | - | - | - | 0.57 | 0.450 |
| rs406193**^ɸ^** |  | 83 | 96.50 | 90.00, 99.00 | 25 | 97.50 | 93.00, 99.00 | 1 | 100.00 | - | - | - | 0.11 | 0.746 |
|  | ZNT5 Site4 | dropped |  |  |  |  |  |  |  |  |  |  |  |  |
|  | ZNT5 Site5 | dropped |  |  |  |  |  |  |  |  |  |  |  |  |
|  | ZNT5 Site6 | dropped |  |  |  |  |  |  |  |  |  |  |  |  |
